# Ten years real-world experience with sacubitril/valsartan in patients with heart failure with reduced ejection fraction

**DOI:** 10.1093/eschf/xvag095

**Published:** 2026-03-31

**Authors:** Gianluigi Savarese, Christian Basile, Alexandre Mebazaa, Antoni Bayes-Genis, Seok Min Kang, Byung-Su Yoo, Carlos Eid, Biykem Bozkurt, Javed Butler

**Affiliations:** Department of Clinical Science and Education, Södersjukhuset, Karolinska Institutet, Sjukhusbacken 10, Stockholm 11883, Sweden; Department of Clinical Science and Education, Södersjukhuset, Karolinska Institutet, Sjukhusbacken 10, Stockholm 11883, Sweden; ANMCO Research Center, Heart Care Foundation, Florence, Italy; Université Paris Cité Hôpital Lariboisière APHP, Paris, France; Hospital Universitari Germans Trias i Pujol, Badalona, Barcelona, Spain; Yonsei University Medical College, Seoul, South Korea; Department of Internal Medicine, Yonsei University Wonju College of Medicine, Wonju, South Korea; Novartis Pharma Services AG, Dubai, United Arab Emirates; Baylor College of Medicine, Houston, TX, USA; Baylor Scott and White Research Institute, Dallas, TX, USA; College of Medicine, University of Mississippi, Jackson, MS, USA

**Keywords:** HFrEF, Sac/Val, Real world, Review

## Abstract

**Introduction:**

Sacubitril/valsartan (Sac/Val) represents a cornerstone of heart failure (HF) with reduced ejection fraction (HFrEF) management. This systematic review provides a comprehensive overview of real-world evidence (RWE) regarding the implementation, clinical effectiveness, and safety of Sac/Val in patients with HFrEF.

**Methods:**

A systematic literature search of PubMed was conducted through March 2024 following PRISMA guidelines.

**Results:**

The review included 45 manuscripts from 30 different studies, primarily from Europe (44%) and the US (30%). RWE confirmed that Sac/Val was associated with a lower risk of cardiovascular mortality (10%-16%), HF hospitalization (10%-38%), and all-cause mortality (10%-25%). Sac/Val was significantly associated with cardiac reverse remodeling and lower-grade mitral regurgitation. Despite these benefits, implementation gaps persist, with only 15%-25% of patients achieving target doses in clinical practice. The most common reported adverse event with Sac/Val was hypotension (up to 17.6%), though severe hyperkalaemia and renal decline were similar when compared with traditional renin angiotensin system inhibitors.

**Conclusion:**

Real-world data mirror the efficacy and safety profiles seen in randomized controlled trials, establishing Sac/Val as a cornerstone of HFrEF therapy. However, significant barriers remain, including delayed initiation and suboptimal dose titration. Enhancing clinician and patient awareness is needed to bridge these implementation gaps and fully realize the drug’s potential to reduce the global healthcare burden of HF.

## Graphical Abstract

**Graphical Abstract:**
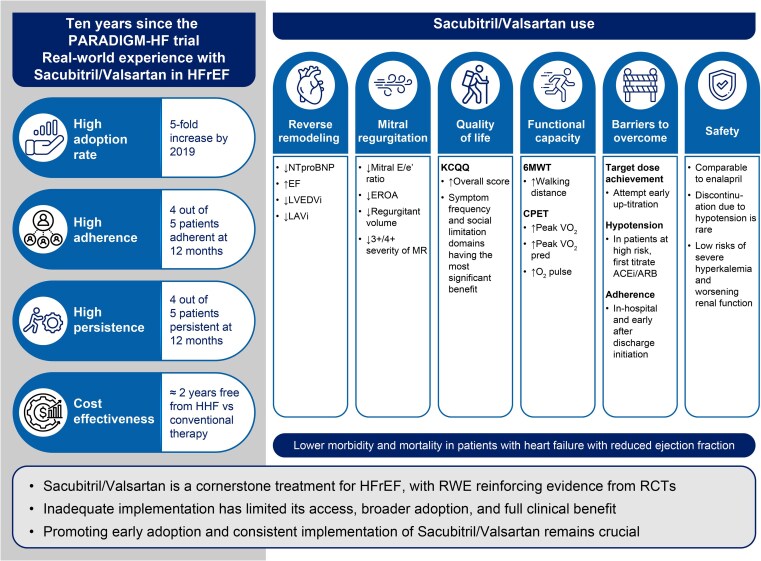
Ten years of real-world evidence on sacubitril/valsartan in heart failure with reduced ejection fraction. 6MWT, six-minute walking test; ACEi, ACE inhibitor; ARB, angiotensin receptor blocker; CPET, cardiopulmonary exercise testing; EF, ejection fraction; EROA, effective regurgitant orifice area; HFrEF, heart failure with reduced ejection fraction; HHF, heart failure hospitalization; LAVi, left atrial volume indexed; LVEDVi, left ventricular end diastolic volume indexed; MR, mitral regurgitation; KCCQ, Kansas City Cardiomyopathy Questionnaire; RCT, randomized controlled trial; RWE, real-world evidence.

## Introduction

Heart failure (HF) is a leading cause of morbidity and mortality worldwide, with an estimated prevalence of 1% to 3%.^1^ Overall, 56.2 million people live with HF globally, and ∼50% of them have HF with reduced ejection fraction (HFrEF, EF ≤40%).^2,3^

Sacubitril/valsartan (Sac/Val), a combination of sacubitril, a neprilysin inhibitor, and valsartan, an angiotensin receptor blocker (ARB), has demonstrated a reduction in cardiovascular (CV) mortality (CVM) or HF hospitalization (HHF), all-cause mortality (ACM) and improved the quality of life (QoL) and decreased symptoms of HF when compared with enalapril in the HFrEF population enrolled in the PARADIGM-HF randomized clinical trial (RCT).^4,5^ Based on these results, regulatory agencies have approved Sac/Val for the treatment of patients with HFrEF.^6–9^ The following RCTs, PIONEER-HF and TRANSITION, reassured about the safety and efficacy of Sac/Val in the in-hospital or early after-discharge settings.^10,11^

Real-world patients with HFrEF frequently differ from those enrolled in RCTs,^12^ wherein patients more likely tolerating and benefiting from the investigated treatment are selected. Moreover, patients’ persistence, adherence, and follow-up frequency differ between clinical practice and the RCT setting. Real-world evidence thus complements evidence from RCTs in a more generalizable setting, providing key insights into the implementation and long-term safety and effectiveness of approved treatments.

This systematic review aimed at providing a comprehensive overview on the real-world experience with Sac/Val on the 10th anniversary of the PARADIGM-HF RCT, by discussing observational real-world findings in the setting of the foundational evidence derived by pivotal randomized controlled trials.

## Methods

A systematic literature search was performed on PubMed until March 2024 (Supplementary Appendix).^13,14^ The quality and risk of bias of the included observational studies were independently evaluated by using the Newcastle-Ottawa Scale for cohort studies (Supplementary Figure S1). Due to the high degree of clinical and methodological heterogeneity among the included studies, particularly regarding study design, comparator, and outcome definition, a quantitative meta-analysis was not feasible, and the systematically retrieved studies were therefore only summarized descriptively.

## Results and discussion

### Study characteristics

**Figure 1:**
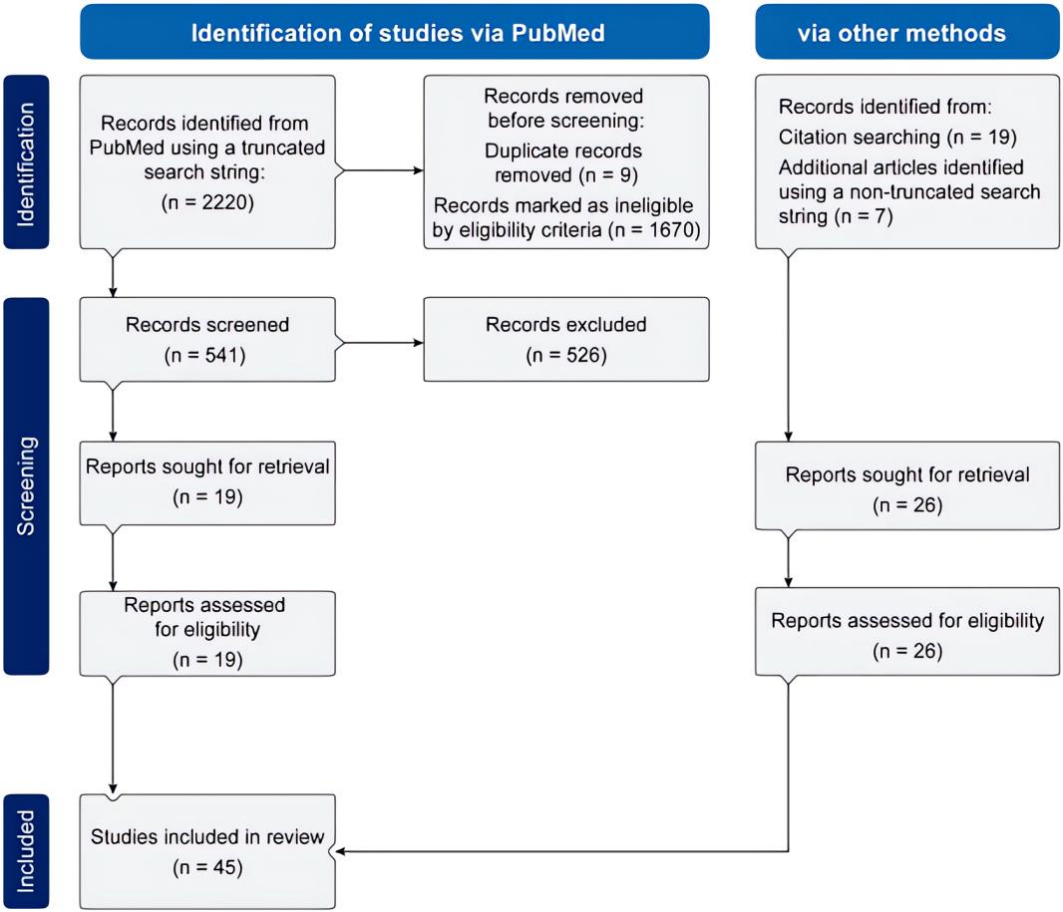
PRISMA flow diagram for literature screening

Supplementary Table S1 presents the main characteristics of the included studies. Of the 2220 manuscripts identified through initial search, 541 were retrieved for more detailed evaluation after removing the duplicates. Thereafter, 45 manuscripts from 30 different studies were included (*Figure 1*). Most of them were conducted in European countries (44%, *n* = 13) and the United States (30%, *n* = 9), with Taiwan (13%, *n* = 4), global (10%, *n* = 3), and Saudi Arabia (3%, *n* = 1) studies being less represented (Supplementary Table S1). The background medications used in the various included studies are presented in Supplementary Table S2. The quality of the included studies is presented in Supplementary Figure S1.

### Efficacy and effectiveness of Sac/Val in HFrEF

#### Reverse remodelling and grading of mitral regurgitation

**Figure 2:**
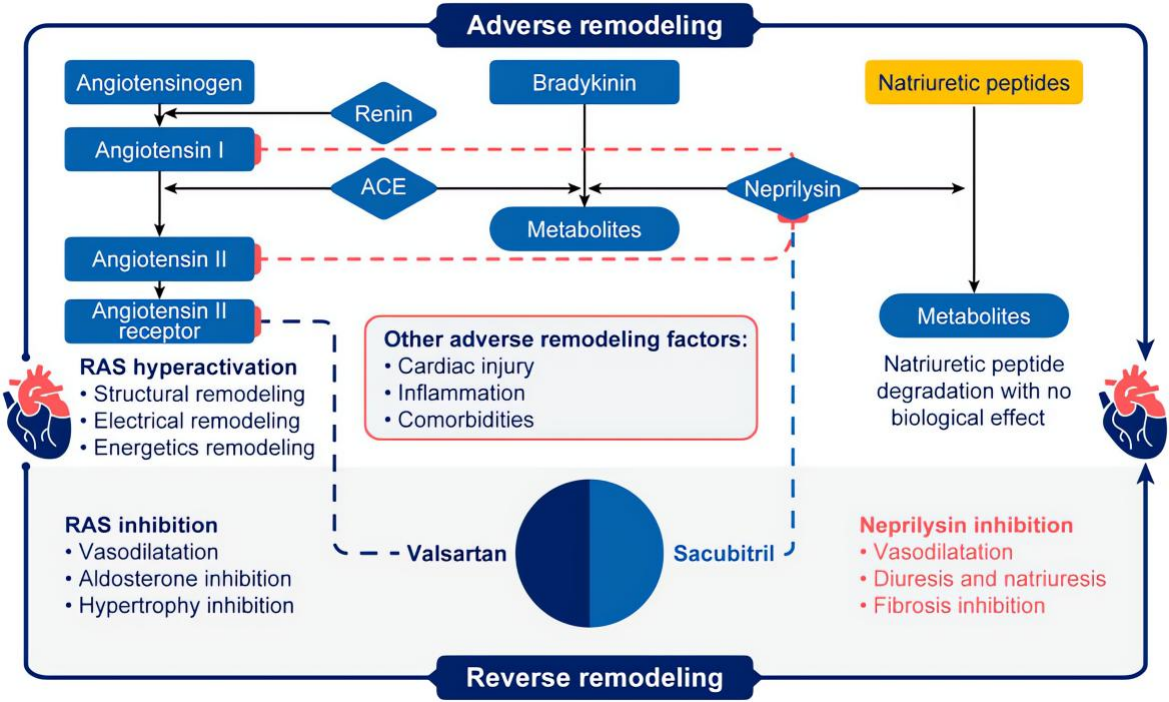
Mechanism of left ventricular adverse and reverse remodelling in patients with heart failure with reduced ejection fraction treated with sacubitril/valsartan. RAS, renin-angiotensin-system

In patients with HFrEF, persistent activation of the renin-angiotensin and sympathetic nervous systems leads to adverse cardiac remodelling, a complex set of maladaptive cellular, metabolic, and extracellular matrix responses resulting in structural and functional changes of the heart,^15^ and consequently increased morbidity and mortality.^16^ Secondary mitral regurgitation (MR) frequently occurs in patients with HFrEF due to cardiac remodelling.^17^ Therefore, reverse remodelling, i.e. the normalization or attenuation of these maladaptive responses, and a reduction in MR, has emerged as an important therapeutic target and outcome in HFrEF (*Figure 2*).

Evidence from both randomized and observational studies suggests a major role for Sac/Val in favouring reverse remodelling. In the EVALUATE-HF RCT, Sac/Val significantly reduced left ventricular and atrial volumes and improved diastolic function when compared with enalapril over a 12-week follow-up.^18^ Similarly, in the PRIME RCT, Sac/Val reduced effective regurgitant orifice area, regurgitant volume, and left ventricular end-diastolic volume and decreased MR when compared with valsartan in patients with HFrEF over a 12-month follow-up period.^19^ Observational studies have confirmed these results. In the PROVE-HF study, 12-month Sac/Val use was associated with higher median EF, lower N-terminal pro-B-type natriuretic peptide (NT-proBNP) levels, smaller left ventricular and atrial volumes, and improved diastolic function when compared with baseline,^20^ with patients achieving lower NT-proBNP levels and smaller left ventricular end-systolic volume indexed at 6 months also experiencing lower rates of 12-month HHF and ACM.^21^ Treatment with Sac/Val was also associated with a lower prevalence of moderate/severe MR at 1 year, with a relative reduction of 44.7%.^22^ Furthermore, patients achieving mild or no MR experienced a higher improvement in EF, lower left ventricular volumes, and NT-proBNP levels at 1 year.^22^ Consistent association between Sac/Val use, lower NT-proBNP levels and MR severity, and higher EF was also reported in other observational studies (*Table 1*).^24–31^ However, these findings should be interpreted critically, as many of these real-world studies on remodelling, such as PROVE-HF and REAL.IT lacked an active control arm and relied on within-patient changes from baseline. Consequently, the observed reverse remodelling may be partially confounded by natural fluctuations in the course of the disease, concurrent optimization of background guideline-directed medical therapy, and the inherent selection bias of patients who survived and adhered to therapy long enough to receive follow-up echocardiograms.

**Table 1:** Studies assessing reverse remodelling following sacubitril/valsartan treatment initiation

	RCT (ref)		Observational studies (ref)								
	EVALUATE-HF^18^ (vs enalapril)	PRIME^19^ (vs valsartan)	PROVE-HF^20–23^	Chang Gung Research Database^24^ (vs ARB)	Cheng Hsin General Hospital^25^	REASSURE^26^	ARNi-TR^27^	REAL.IT study^28^	Mapelli et al^29^	IRRB/23/15^30^	Campanile et al^31^ (vs ACEi/ARB)
**Number of patients**	464 (overall), 231 (Sac/Val)	118 (overall), 60 (Sac/Val)	794 (Sac/Val)	991 (overall), 502 (Sac/Val)	437 (Sac/Val)	600 (Sac/Val)	779 (Sac/Val)	924 (Sac/Val)	97 (Sac/Val)	134 (Sac/Val)	25 (overall), 12 (Sac/Val)
**Follow-up (months)**	3	12	12	12	12	12	12	12	10.1 (2.2)	12	16 [11.5-22]
**NT-proBNP, pg/mL**	Ratio of geometric means vs baseline: 0.63 (95% CI 0.58–0.69) Ratio of geometric means vs enalapril: 0.67 (95% CI 0.59–0.76)	NA	Change vs baseline: −37% (*P* < .001)	NA	NA	Ratio of geometric means vs baseline: 0.5 (95% CI: 0.4–0.5)	Baseline: 1487 [609–3277] Follow-up: 633 [327–1594], *P* < .001	−20% (*P* < .001)	Baseline: 1179 [610–2757] Follow-up: 780 [372–1344], *P* < .001	Baseline: 1443.2 (1323) Follow-up: 1041.2 (1171.3), *P* = .015	NA
**Ejection fraction, %**	Change vs baseline: 1.9 (95% CI: 1.2–2.6). Change vs enalapril: 0.6 (95% CI: −0.4, 1.7)	NA	Change vs baseline: 9.4 (95% CI: 8.8–9.9)	Follow-up: Sac/Val 39.1 (13.8) ARBs 39.9 (14.4), *P* = .29	Change vs baseline: 7.7 (11.1), *P* < .001	Change vs baseline: 10.4 (12.2)	Baseline: 28.0 (6.5) Follow-up: 30.8 (7.5), *P* < .001	Baseline: 28.7 (5.8), Follow-up: 32.8 (7.9), *P* < .001	Baseline: 31.4 (4.7) Follow-up: 36.6 (8.1), *P* < .001	Baseline: 28 (5.8) Follow-up: 31.8 (7.3), *P* < .001	Follow-up: Sac/Val 36.1 (14.8) ACEi/ARB 33.1 (7.8), *P* = .06
**Left ventricular end-diastolic volume indexed, mL/m^2^**	Change vs baseline: −5.2 (95% CI: −6.4, −3.9). Change vs enalapril: −2.0 (95% CI: −3.7, −0.3)	Change vs baseline: −11.8 (17.3). Change vs valsartan: −7.01 (95% CI: −13.83, −0.19)	Change vs baseline: −12.2 (95% CI: −12.9, −11.6)	NA	NA	Change vs baseline: −18.7 (26.1)	NA	NA	Baseline: 94 [79–114) Follow-up: 84 [67–98], *P* < .001	Baseline: 112.2 (27.9), Follow-up: 112.9 (25.8), *P* = .897	Follow-up: Sac/Val 80.3 (19.1) ACEi/ARB 125.0 (37.2), *P* = .09
**Left ventricular end-diastolic diameter (mm)**	NA	NA	NA	Follow-up: Sac/Val 59.0 (10.1) ARBs 59.2 (10.5), *P* = 0.76	Change vs baseline: −2.2 (5.5), *P* < .001	Change vs baseline: −4.5 (5.9)	NA	NA	NA	Baseline: 63.1 (6.7) Follow-up: 63.6 (6.5), *P* = .701	NA
**Left atrial volume index, mL/m^2^**	Change vs baseline: −2.2 (95% CI: −3.0, −1.3). Change vs enalapril: −2.8 (95% CI: −4.0, −1.6).	NA	Change vs baseline: −7.6 (95% CI: −8.0, −7.1)	NA	NA	NA	NA	NA	Baseline: 44.3 [34.8–56.8) Follow-up: 37.9 [33.0–51.3], *P* < .001	Baseline: 49.6 (17.9) Follow-up: 45.6 (14.7), *P* = .226	NA
**Mitral E/e′ ratio**	Change vs baseline: −1.4 (95% CI: −2.1, −0.7). Change vs enalapril: −1.8 (95% CI: −2.8, −0.8).	NA	−1.3 (95% CI: −1.7, −0.9)	NA	NA	NA	NA	NA	NA	Baseline: 15.1 (6.8) Follow-up: 13.4 (7.0) *P* = .298	Follow-up: Sac/Val 1.3 (1.0) ACEi/ARB 1.4 (1.0) *P* = .07
**EROA of MR, cm^2^**	NA	Change vs baseline: −0.058 (0.095) Change vs valsartan: −0.040 (95% CI: −0.076, −0.094).	NA	NA	NA	NA	NA	NA	NA	NA	NA
**Regurgitant volume, mL**	NA	Change vs baseline: −11.6 (14.4) Change vs valsartan: −7.3 (95% CI: −12.6, −1.9)	NA	NA	NA	NA	NA	NA	NA	NA	NA
**MR 3+/4+**	NA	NA	Change vs baseline: −44.7%	Follow-up: Sac/Val 9.0% ARBs 13.6%, *P* = .06	Baseline: 31.8% Follow-up: 23.8%, *P* < .001	NA	NA	NA	NA	NA	NA

Values are mean (standard deviation) or median [interquartile range] if not otherwise specified.

ACEi, ACE inhibitor; ARB, angiotensin receptor blocker; CI, confidence interval; EROA, effective regurgitant orifice area; MR, mitral regurgitation; NA, not available; NT-proBNP, N-terminal pro B-type natriuretic peptide; RCT, randomized controlled trial; Sac/Val, sacubitril/valsartan.

#### Implantable cardioverter defibrillators

Implantable cardioverter defibrillators (ICDs) are recommended for the primary and secondary prevention of life-threatening arrhythmias and sudden cardiac death in patients in the lower spectrum of EF (EF ≤35%) after at least 3 months of optimal medical therapy.^32,33^ Considering its role in reverse remodelling, Sac/Val may reduce the need for ICD implantation. When only patients eligible for an ICD were considered in the PROVE-HF study, of the 661 patients initially eligible at baseline, 32% and 62% were no longer eligible (EF >35%) at 6 months and 12 months, respectively.^34^ In the DISCOVER-ARNi study, 60% of the 126 patients enrolled were no longer eligible for ICD implantation after 6 months of treatment with Sac/Val.^35^ Similar results were reported in smaller studies from Italy and Portugal, showing that 40% and 56% of patients were no longer eligible for ICD after a median follow-up of 11 months, respectively.^36,37^

#### Quality of life

QoL is as important as survival to most patients living with chronic illnesses such as HFrEF.^38^ QoL reflects the multidimensional impact of a disease and its treatment on a patient’s daily life, with poor QoL also associated with high hospitalization and mortality rates.^39,40^

Sac/Val use improved the QoL in both RCTs and observational studies (*Table 2*). In the PARADIGM-HF RCT, Sac/Val improved patient-reported QoL,^5^ with higher Kansas City Cardiomyopathy Questionnaire-overall scores (KCCQ-OS) than enalapril, and the greatest improvements were observed in household chores and sexual relationships.^41^ In observational studies, the CHAMP-HF registry showed a significant association between Sac/Val initiation and higher KCCQ-OS at 2 months when compared with no Sac/Val initiation, with more patients on Sac/Val reporting large (≥10-point) and very large (≥20-point) gains in KCCQ-OS (32.7% vs 26.9% and 20.5% vs 12.1%, respectively).^42^ These results were consistent regardless of ethnicity and at a later follow-up, i.e. 18 months after treatment initiation.^43,44^ Interestingly, the most significant benefit was seen in the QoL symptom frequency and social limitation domains, as in the PARADIGM-HF trial.^41,42^ Consistent results were observed in the PROVE-HF study, wherein Sac/Val initiation was associated with a higher KCCQ-OS score: 56.2% of patients achieved a large (≥10-point) and 33.4% a very large (≥20-point) gain in KCCQ-OS at 12 months compared with baseline.^23^ While these real-world data mirror the benefits in terms of symptom burden mitigation seen in trials, it is important to acknowledge that assessing patient-reported outcomes in open-label, observational settings might introduce potential placebo effects and recall bias. Furthermore, patients who experience significant adverse events or fail to improve may be more likely to drop out of voluntary registries, potentially leading to overestimate the overall quality-of-life benefits in the broader population.

**Table 2:** Studies assessing quality of life following sacubitril/valsartan treatment initiation

Study (ref)	Number of patients	Age, female %	Follow-up (months)	Measure of quality of life	Overall change vs baseline value	Overall change vs comparator	Domain with greatest change
**PARADIGM-HF (RCT)** ^5,41^	8399 (overall); Sac/Val: 3797	64 years; 21%	Up to 36	KCCQ-23	NA	Overall KCCQ-OS: 1.19 (0.28); *P* < .001 Overall KCCQ-CS: 0.99 (0.28); *P* < .001	Household chores (overall change score difference, 1.69; *P* < .001) and intimate relations (overall change score difference, 2.36; *P* < .001)
**PROVE-HF** ^23^	794	64–66 years; 25–34%	12	KCCQ-23	48–196 mg daily dose: 68.80 vs 63.12 200–371 mg daily dose: 72.50 vs 61.42 372–400 mg daily dose: 73.11 vs 63.76	NA	NA
**CHAMP-HF** ^42–44^	∼4000 (overall); Sac/Val: ∼760	64 years; 30%	Up to 18 months	KCCQ-12	64.1 (23.5) in Sac/Val vs 63.3 (23.9) in no-Sac/Val^42^	Sac/Val: (5.3 ± 19 vs no-Sac/Val: 2.5 ± 17.4, respectively; difference: 3.2 (95% CI: 1.5, 4.9) *P* < .001)^42^ No significant interaction between race and ethnicity and ARNi initiation^44^	Very large improvement (≥ 20) in QoL domain in 25.6% of patients^43^

CS, clinical summary; KCCQ, Kansas City Cardiomyopathy Questionnaire; OS, overall summary.

#### Functional capacity

Exercise intolerance is a hallmark feature of HFrEF.^45^ Functional capacity, i.e. the ability to perform exercise, is usually assessed by using the 6-minute walk test (6MWT) or cardiopulmonary exercise testing (CPET) and is significantly impaired in patients with HFrEF.^33^

In the OUTSTEP-HF RCT, Sac/Val showed no significant benefit in functional capacity, as measured by the 6MWT, compared with enalapril at 12 weeks.^46^ Consistent results have been observed in observational studies. In a small prospective study enrolling 58 patients with HFrEF, Sac/Val initiation was associated with a 13.9% longer distance at the 6MWT at 30 days vs baseline.^47^ When CPET was used to assess functional capacity, Sac/Val initiation was associated with a dose-dependent improvement in functional capacity in terms of an increase in O_2_ intake at peak exercise and a reduction in the minute ventilation/carbon dioxide production slope when compared with baseline values,^29–31,48,49^ despite no difference observed vs the comparator (*Table 3*).^31^ These real-world data should be interpreted while also taking into account potential limitations. The studies are largely limited by small sample sizes and limited adjustment for confounding. Furthermore, because both 6MWT and CPET are highly effort-dependent, the open-label design might introduce a performance bias; patient and investigator expectations may positively bias exercise results, making it difficult to definitively isolate the pharmacological effects of Sac/Val from placebo effects or general care optimization.

**Table 3:** Randomized controlled trial and observational studies assessing the association between sacubitril/valsartan and functional capacity

Study (ref)	Number of patients	Age, female %	Follow-up (months)	Measure of functional capacity	Difference with baseline value	Difference with comparator
**OUTSTEP-HF (RCT)** ^46^ **(vs enalapril)**	619 (overall), 309 (Sac/Val)	67.2 (11.4) years, 23% females	3	Six-minute walking distance	35.09 meters (97.5% CI: 27.85, 42.32)	8.98 meters (97.5% CI: −1.31, 19.27)
**Beltrán et al** ^47^	58 (Sac/Val)	70 (11) years, 72.4% females	1	Six-minute walking distance	41.8 meters (33.4–50.2)	NA
**Mapelli et al** ^29^	97 (Sac/Val)	63.7 (9.8) years, 20% females	10.1 (2.2)	CPET	Peak VO_2_ (mL/min/kg): from 15.6 (4.5) to 16.5 (4.9), *P* < .001 Peak VO_2_ (% pred): from 63 (16) to 68 (17), *P* < .001 O_2_ pulse (mL/b): from 11.9 (4.1) to 12.1 (3.5), *P* < .001	NA
**IRRB/23/15** ^30,48,49^	134 (Sac/Val)	57.9 (9.6) years, 13% females	12	CPET	Peak VO_2_ (mL/min/kg): from 15.3 (3.7) to 17.8 (4.2), *P* < .001 Peak VO_2_ (% pred): from 56.4 (13.9) to 64.8 (17.8), *P* < .001 O_2_ pulse (mL/b): from 11.4 (3) to 13.7 (4.6), *P* < .001	NA
**Campanile et al.** ^31^ **(vs ACEi/ARB)**	25 (overall), 12 (Sac/Val)	66.1 (7.9) years, 16.7% females	16 [11.5, 22]	CPET	Peak VO_2_ (mL/min/kg): from 12.2 (4.6) to 12.7 (3.3) Peak VO_2_ (% pred): from 61.5 (25.7) to 67.0 (23.7)	Follow-up Peak VO_2_ (mL/min/kg): Sac/Val 12.7 (3.3) ACEi/ARB 13.0 (4.2) *P* = .49 Follow-up Peak VO_2_ (% pred): Sac/Val 67.0 (23.7) ACEi/ARB 61.1 (23.9) *P* = .53

Values are mean (standard deviation) or median [interquartile range] if not otherwise specified.

ACEi, ACE inhibitor; ARB, angiotensin receptor blocker; CI, confidence interval; CPET, cardiopulmonary exercise testing; NA, not available; NT-proBNP, N-terminal pro B-type natriuretic peptide; RCT, randomized controlled trial; Sac/Val, sacubitril/valsartan.

#### Sac/Val and mortality/morbidity in patients with HFrEF

In the PARADIGM-HF trial, Sac/Val showed a 20% reduction in the composite primary endpoint of CVM or HHF vs enalapril. Sac/Val also reduced CVM (20%), HHF (21%), and ACM (16%).^4^ Since the approval of Sac/Val for the treatment of HFrEF, several observational studies, as well as systematic reviews and meta-analyses of real-world data have confirmed its effectiveness, with the use of Sac/Val being associated with a lower risk of CVM (10%–16%), HHF (10%–38%), and ACM (10%–25%).^50–52^ Although these findings do not rely on clinically adjudicated endpoints but rather on outcomes defined according to ICD-10 codes which could result in misclassification, and might also be affected by unmeasured confounding and bias that prevent causal inference in observational studies, the results concerning the effectiveness of Sac/Val align with the efficacy previously demonstrated in RCTs.

### Implementation of Sac/Val in clinical practice: current challenges and gaps in clinical practice

Despite the efficacy of medical therapies, HFrEF prognosis remains poor, largely due to challenges in treatment use and implementation in routine clinical practice.

#### Limited use and target dose achievement

The adoption of Sac/Val by physicians has increased over time. A retrospective cohort study from the Veterans Affairs registry performed within the first 2 years of the US Food and Drug Administration approval showed that only a minority of patients (4.2%) switched from an angiotensin-converting enzyme (ACE) inhibitor (ACEi)/ARB to Sac/Val.^53^ In later analyses from US prescription databases, the prescription of Sac/Val was shown to have been increased fivefold by 2019 (*Table 4*).^54,55^

**Table 4:** Implementation of Sac/Val in clinical practice: use and target dose achievement

Study name	Country (Dataframe)	Timeframe	Prescription	Result(s)
**Mohanty et al** ^53^	US (Veterans Affair)	2015–2017	Switching from ACEi/ARB to Sac/Val	Only 4.2% of eligible patients switched
**Ozaki et al** ^54^	US (National Prescription Audit^TM^ data)	2016–2019	Sac/Val use and dosage patterns	5.6-fold increase in Sac/Val prescriptions 14% of eligible patients switched 24/26 mg: 48.7% 97/103 mg: 20.6%
**Sumarsono et al** ^55^	US (Medicare Part D and Medicaid)	2016–2017	Sac/Val prescriptions	Utilization trends increased by 156–251%
**Stolfo et al** ^56^	Sweden (HF registry)	2017–2021	Sac/Val initiation	8.3% (2017) 26.7% (2021)
**Savarese et al** ^57^	Sweden (HF registry)	Patients since 2000	Eligibility for Sac/Val	57% had EF <40%; Sac/Val eligibility 67% (pragmatic criteria) and 38% (literal trial criteria)
**Mohebi et al** ^23^	US (PROVE-HF study)	12 months of treatment	Dose-response to Sac/Val	Similar reduction in stress biomarkers, similar improvement in health status, and comparable reversal in cardiac remodeling process across all 3 dose categories
**D'Amario et al** ^58^	Sweden (HF registry)	2000–2018	Association between number and dosing of GDMT	46% had ≥100% of target dose achievement for ACEi/ARB/ARNi 22% had ≥100% of target dose achievement for ACEi/ARB/ARNi + beta-blocker
**Wachter et al** ^59^	Germany (IMS® longitudinal prescriptions database)	2016–2017	Sac/Val treatment patterns	Two-thirds of patients were prescribed the lowest Sac/Val dose at index. > 80% of the patients remained on the higher uptitrated dose following uptitration
**Park et al** ^26^	Korea (patient-level medical records)	2017–2019	Sac/Val treatment patterns	Stable uptitration’ (41.5%) At 12 months: 24.8% of target doses
**Peri-Okonny et al** ^60^	US (CHAMP-HF registry)	2015–2017	Use of target doses of foundational GDMT	<20% of patients were receiving target doses

Slow implementation was also initially observed in Europe. A recent study from the Swedish HF registry showed that Sac/Val use increased from 8.3% in 2017 to 26.7% in 2021.^56^ When eligibility for Sac/Val was considered using the same data source, 67% of patients with HFrEF would have qualified for Sac/Val according to the pragmatic scenario, which adopts the criteria used in daily clinical practice.^57^ Eligible patients tended to be older, more likely female, with more severe HF and a higher burden of comorbidities.^57^ Interestingly, event rates for ACM, CVM, and HHF were higher for eligible vs non-eligible patients, further suggesting the significant impact that Sac/Val implementation might have in this eligible but untreated population.^57^

In the PROVE-HF and PIONEER-HF studies, Sac/Val provided similar reductions in NTproBNP regardless of the achieved dose.^23,61^ In the PARADIGM-HF RCT, the relative benefit with Sac/Val at lower doses vs lower doses of enalapril was similar to that achieved with target doses of Sac/Val vs enalapril. However, dose reduction was associated with a higher incidence of the primary outcome.^62^ The achievement of target doses of HF medications, including Sac/Val, has been associated with lower morbidity and mortality in HFrEF,^58^ further highlighting the need for uptitrating doses aiming at achieving the target dose when patients can tolerate them. However, these observational associations should be interpreted taking into consideration potential confounding and bias related to treatment tolerance, as patients who achieve higher doses are likely to represent a healthier, more haemodynamically stable subgroup.

In an analysis of 12 082 patients receiving Sac/Val from the German pharmacy prescription database, two-thirds of the patients were prescribed the lowest dose at index, with uptitration attempted in only 41% of these patients during the first 6 months of follow-up. However, when uptitration was attempted, >80% of the patients remained on the higher uptitrated dose, with the target dose ultimately achieved in 21% of patients. Patients treated with Sac/Val also showed high adherence and persistence to therapy, with 71% persistence and 81% proportion of days covered (PDC) at 12 months.^59^ Consistent results were reported in a Korean cohort, with 60% of patients starting on the lowest dose, uptitration attempted in 41.5% of patients, and target dose achieved in 24.8% of patients by month 12,^26^ and in the CHAMP-HF, where the target dose was achieved in 15% of patients, with the majority (52%) being treated with the lowest dose.^60^

Overall, these data indicate that despite increased use of Sac/Val, many eligible patients remain untreated, and achieving optimal dose remains a significant challenge. This underscores the need for targeted efforts to improve prescription rates and dose titration to fully realize the clinical benefits of Sac/Val in patients with an indication for treatment.

#### De novo and in-hospital initiation of Sac/Val

In the PARADIGM-HF RCT, randomization to Sac/Val or enalapril was performed after a run-in period with enalapril and then Sac/Val.^4^ This specific feature of the PARADIGM-HF design has led the European HF guidelines to recommend (class I) Sac/Val as a replacement to ACEi in patients who remain symptomatic despite optimal medical therapy, or its *de novo* use in naïve ACEi patients based on the results of the PIONEER-HF and TRANSITION trials,^10,11^ albeit with a lower class of recommendation (class IIb).^33^ The ACC/AHA/HFSA guidelines recommend Sac/Val with CoR I, level of evidence A for all patients with HFrEF (class 1A), irrespective of previous therapy.^32,63^

In the real world, *de novo* use of Sac/Val ranged from 23% in the Swedish HF registry to 26.5% and 33.7% in analyses of the Veterans Health Affairs database and tertiary care hospitals in Saudi Arabia, respectively.^64–66^ In the GWTG-HF registry, when patients hospitalized for HF and eligible for Sac/Val were considered, 4.1% initiated Sac/Val as inpatients and 2.8% at discharge.^67^ Discharge without any prescription for Sac/Val was an independent predictor of not receiving the treatment over 1-year follow-up.^67^ A combined analysis of United States/United Kingdom/Sweden administrative databases considering new users of HF medical therapy following an HHF showed that of 29 546 Sac/Val users, 22% were *de novo* users.^68^ Within 1 year after the treatment initiation, the target dose of Sac/Val was achieved in 30% of patients on treatment, with a 27% of discontinuation rate. Only 5.7% and 6.6% of patients initiated on ACEi and ARB started Sac/Val during the 1-year follow-up, respectively.^68^ Sac/Val has been shown to be initiated at lower doses in the in-hospital vs outpatient setting,^69^ with in-hospital and early initiation quite limited (8%).^56^ Sac/Val was more likely initiated later than earlier during the disease course and in patients with more severe HF, which might suggest its use as a second-line treatment after clinical deterioration. Discontinuation rates were consistent across initiation settings.^56,69^ In the EVOLUTION-HF study, combining 266 589 patients with HFrEF from US, Japan, and Sweden, the mean time from HHF to therapy initiation was longer for newer treatments, i.e. dapagliflozin and Sac/Val, when compared with other HF therapies [39 and 44 vs 12–13 days (Japan), 44 and 33 vs 22–31 days (Sweden), and 33 and 19 vs 18–24 days (US)].^70^ Lastly, in a retrospective US cohort study using electronic health records and including ACEi/ARB-naïve patients (*N* = 6118) with HFrEF, *de novo* Sac/Val initiation (*N* = 3059) was associated with a significantly lower risk of HHF or emergency room visits than ACEi/ARB initiation [hazard ratio (HR): 0.92; *P* = .01].^71^ Despite the low use of *de novo* Sac/Val, likely due to the fear of adverse events (hypotension, angioedema) or the lower class of recommendation in the guidelines, the risk of angioedema was lower among >40 000 Sac/Val new users (HR: 0.18) than among previous ACEi/ARB users in an analysis from the Sentinel Distributed Database.^72,73^

**Figure 3:**
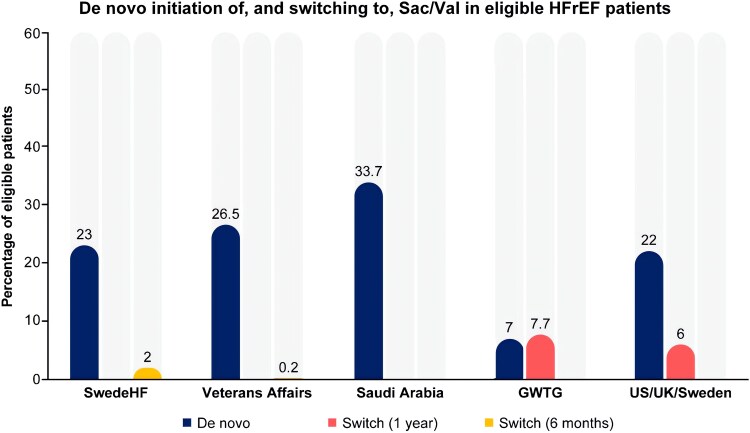
De Novo and in-hospital initiation of Sac/Val

Overall, these data support the safety and effectiveness of *de novo* and in-hospital initiation of Sac/Val, especially given the similar persistence rates and the lower Sac/Val use at 1 year in patients transitioning from ACEi/ARB vs *de novo* Sac/Val users (*Figure 3*).

### Perceived barriers to the implementation of Sac/Val

**Figure 4:**
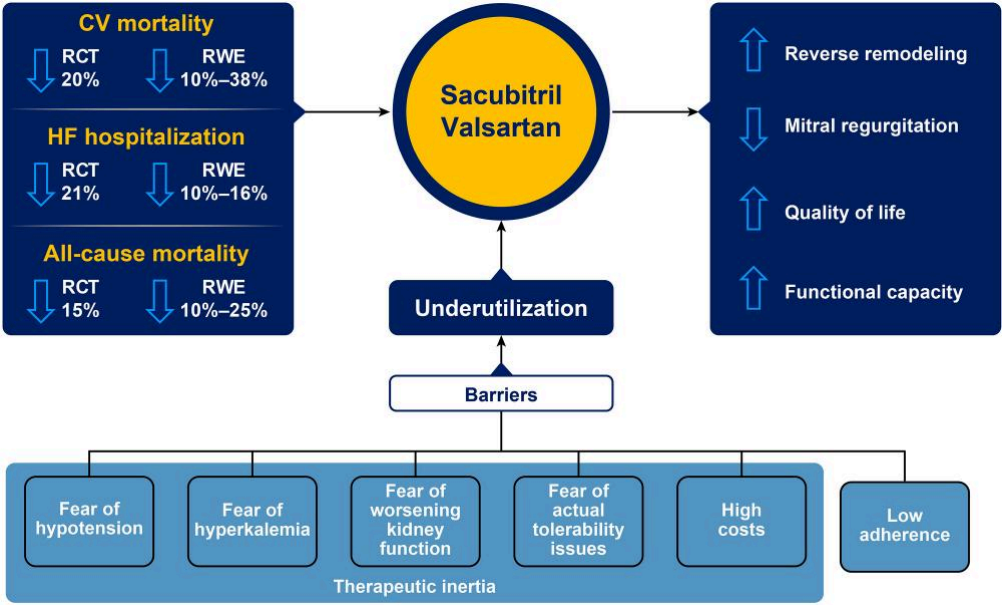
Factors limiting the implementation of sacubitril/valsartan. RCT, randomized controlled trial; RWE, real-world evidence

Despite its proven efficacy and safety, Sac/Val remains underutilized in clinical practice due to real or perceived patient- and physician-related barriers (*Figure 4*).^74,75^

#### Patient´s adherence

Patients treated with Sac/Val (*n* = 22 275) showed high adherence and persistence to therapy, with 71% of persistence and 81% of PDC at 12 months in an analysis of the German pharmacy prescription database.^59^ A retrospective analysis of the Medicare claims database showed that a good 6-month adherence, defined as PDC ≥80%, was observed in 59.1% of patients (*n* = 1161), with half of the subgroup of patients with low adherence (PDC <80%) discontinuing Sac/Val within 6 months of initiation.^76^ Black ethnicity was associated with limited adherence, while treatment with other HF medications, previous treatment with ACEi/ARB, and initiation with the target dose were associated with high adherence.^76^ Adherence was also reported to be high in an analysis of patients (*n* = 8291) treated with Sac/Val from US administrative claims databases, showing a medical possession ratio of 94% over a median follow-up of 4.8 months, and patients of Black ethnicity having lower adherence than patients of White ethnicity.^77^ Interestingly, an analysis of 897 patients from the GWTG-HF registry hospitalized for HF and discharged on Sac/Val showed that higher adherence to Sac/Val was associated with lower 90-day and 1-year all-cause hospitalization and ACM than lower adherence.^78^ More recently, a nationwide longitudinal cohort study (PARADE-HF) investigated medication adherence with Sac/Val (13 483 patients) vs ACEi/ARBs (13 483 patients) in patients with HFrEF. The risk reduction on 1-year all-cause mortality/hospitalization for Sac/Val vs ACEi/ARBs was higher in patients with PDC ≥80%, but not in those with PDC <80%.^79^

Overall, these data indicate that the treatment with Sac/Val is characterized by high adherence, which is associated with improved clinical outcomes. Disparities, such as lower adherence in Black patients, highlight the need for targeted strategies to ensure equitable benefits of therapy in all patients.

#### Safety: hyperkalaemia, hypotension, and declining kidney function

Data from the PARADIGM-HF RCT highlighted a comparable safety profile for Sac/Val vs enalapril.^4^ Although patients on Sac/Val more likely experienced symptomatic hypotension, discontinuation due to hypotension was infrequent.^4^ During the enalapril and Sac/Val run-in phases of the trial, hypotension occurred in 1.3% (*n* = 136) and 2.4% (*n* = 228) of the 10 513 patients, leading to discontinuation in 68% and 51% of these cases, respectively.^80^ Although hypotension during run-in was relatively uncommon, patients on subtarget doses for ACEi/ARBs had a higher risk (4% vs 3%).^80^ This, together with patients experiencing hypotension having lower systolic blood pressure at screening,^80^ may suggest that careful uptitration of renin-angiotensin system inhibitors before initiating Sac/Val may help mitigate the risk of hypotension in vulnerable patients. Additionally, in the PARADIGM-HF, patients on Sac/Val who also experienced a significantly slower decrease in estimated glomerular filtration rate (eGFR) over time were less likely to experience severe hyperkalaemia (potassium levels >6.0 mEq/L),^81,82^ but were more likely to be on mineralocorticoid receptor antagonist (MRA) therapy when compared with patients randomized to enalapril.^83^ In the PIONEER-HF, the occurrence of worsening renal function, hyperkalaemia, symptomatic hypotension, and angioedema was comparable between the enalapril and Sac/Val arms,^10^ with low discontinuation rates due to adverse events (AEs) for Sac/Val also shown in the TRANSITION RCT.^11^

Similar to the aforementioned RCTs, observational studies also identified hypotension, hyperkalaemia, and worsening kidney function as the most frequent AEs with Sac/Val treatment.^20,27,28,77^ A propensity score-matched analysis of US administrative claims databases found a 35% significantly higher risk of hypotension with Sac/Val when compared with enalapril (3.4 vs 2.5 events per 100 person-years), but no significant difference in the risk of hyperkalaemia (0.89 vs 0.84 events per 100 person-years).^77^ The REAL.IT study reported no significant change in potassium levels after 12 months of Sac/Val treatment when compared with baseline, although a very modest decline in mean eGFR (68 vs 65 mL/min/m^2^) was observed.^28^ The ARNi-TR study also showed a small reduction in median eGFR at 12 months after Sac/Val initiation, alongside a slight increase in median potassium levels (4.2 vs 4.4 mmol/L).^27^ Importantly, both the REAL.IT and ARNi-TR studies did not have a control arm, and therefore changes in renal function might reflect the usual decline in renal function over time observed in patients with HF, rather than any phenomenon related to the drug. Lastly, in the PROVE-HF study in 794 patients, hypotension (17.6%), hyperkalaemia (13.2%), and worsening kidney function (12.3%) were the most frequently reported AEs after 12 months of Sac/Val use.^20^ When examined by setting, rates of AEs were similar for both inpatients and outpatients initiating Sac/Val [hypotension: 16.0 (*n* = 16) vs 16.7% (*n* = 71); worsening renal function: 7.0 (*n* = 7) vs 6.8% (*n* = 29); hyperkalaemia: 1.0 (*n* = 1) vs 4.9% (*n* = 21), none *P* < .05].^69^

A disproportionality analysis of 103 038 AEs from the VigiBase, FAERS, and EudraVigilance pharmacovigilance databases showed that Sac/Val had the greatest association with hypotension (odds ratio: 11.4) when compared with other HF treatments.^84^ In contrast, hyperkalaemia, renal dysfunction, and angioedema were more likely associated with spironolactone, enalapril, and lisinopril, respectively.^84^

In the LIFE trial, an RCT testing Sac/Val vs valsartan after a first run-in phase in patients with advanced HFrEF, similar rates of discontinuations were observed during the trial in patients randomized to Sac/Val [29% (*n* = 49)] or valsartan [21% (*n* = 36), *P* = .1], with no significant difference in incidence of hypotension or worsening kidney function between the treatment arms.^85^ Significantly more patients developed hyperkalaemia with Sac/Val (17% vs 9%, *P* = .04).^85^ An analysis assessing clinical parameters associated with run-in failure in the LIFE trial showed that of all the patients entering the run-in phase (445 subjects), only a minority was intolerant to Sac/Val [18% (*n* = 73)].^86^ Lower mean arterial pressure, serum chloride, the presence of an ICD and/or cardiac resynchronization therapy, moderate or greater MR, nonuse of ACEi/ARB at screening, and use of insulin were all independently associated with a higher likelihood of run-in failure.^86^ When a similar analysis was performed in the PARADIGM-HF RCT, lower systolic blood pressure, eGFR, higher NT-proBNP levels, and an ischaemic cause of HF were associated with a higher risk of run-in noncompletion (19.8% of all patients entering the run-in phase).^87^

Overall, these data suggest that hypotension is more likely observed in patients on Sac/Val, particularly in more vulnerable subgroups. The risk of severe hyperkalaemia and worsening renal function appears lower than with ACEi/ARBs, which is important as these are the main perceived barriers to Sac/Val implementation.^88^

#### Cost-effectiveness

Evaluating the cost-effectiveness of medical therapies is necessary for value-based decision-making. An early analysis of a retrospective Medicare claims database showed that the adoption of Sac/Val was slow due to high cost concerns.^76^

Decision-analytic Markov model showed that Sac/Val use was associated with a relative increase of 0.04 quality-adjusted life-years (QALY) and 0.67 years of life lived per person compared with the standard of care, with cost-effectiveness maintained in ≥96.5% of simulated cases on the outcomes of ICD implantation and ACM.^89^ When combination therapy was considered, HF quadruple therapy with Sac/Val (Sac/Val, beta-blocker, MRA, and sodium glucose cotransporter 2 inhibitors) resulted in an increase of 1.73 life-years vs triple HF therapy (ACEi, beta-blocker, and MRA) and double therapy (ACEi, beta-blocker), corresponding to an increase of 1.12 years and 1.85 years in QALYs, resulting in a cost-effectiveness of 91.7% and 99.9%, respectively, in analyses considering the PARADIGM-HF RCT and the Spanish National Health Service, respectively.^90–92^ These findings suggest the cost-effectiveness of Sac/Val, owing to the lower rates of hospitalizations and mortality. The combination of Sac/Val, beta-blocker, and MRA was estimated to provide an additional 8.3 years free from CVM or hospitalizations when compared with 6.3 years when considering conventional ACEi/ARB and beta-blocker therapy.^93,94^

### Limitations

Although a total of 45 articles were included, the number of studies addressing specific endpoints was lower, limiting the applicability of our results. This systematic review focused exclusively on the real-world experience with Sac/Val in patients with HFrEF, with Sac/Val being approved for adult patients other than HFrEF only in the US and Japan. Other limitations include the limited availability of data on trends in Sac/Val utilization over time, and on the effects of Sac/Val in patients with pulmonary hypertension or right ventricular failure. Furthermore, there is a consistent underrepresentation of women across both the pivotal clinical trials and the real-world cohorts included in this systematic review. This sex imbalance highlights a significant gap in the literature, and may limit the generalizability of these findings to women with HFrEF. Additionally, the inherent bias in some of the included studies may limit the generalizability of the findings.

## Conclusions

Sac/Val is a cornerstone treatment for HFrEF, with real-world evidence over the past decade reinforcing the benefits observed in RCTs (Graphical abstract). However, inadequate implementation has limited its access, broader adoption, and full clinical benefit. Addressing these gaps by increasing clinicians’ and patients’ awareness is crucial for promoting earlier adoption and consistent implementation of Sac/Val in routine care, thereby ensuring that eligible patients fully benefit from the best possible standard of care.

## Supplementary Material

xvag095_Supplementary_Data

## Declarations

### Data availability

No data were generated or analysed for this manuscript.

### Pre-registered clinical trial number

None supplied.

## References

[1] Savarese G, Becher PM, Lund LH, Seferovic P, Rosano GMC, Coats AJS. Global burden of heart failure: a comprehensive and updated review of epidemiology. Cardiovasc Res 2023;118:3272–87. 10.1093/cvr/cvac013 35150240

[2] GBD 2019 Diseases and Injuries Collaborators . Global burden of 369 diseases and injuries in 204 countries and territories, 1990-2019: a systematic analysis for the Global Burden of Disease Study 2019. Lancet 2020;396:1204–22. 10.1016/S0140-6736(20)30925-9 33069326 PMC7567026

[3] Bozkurt B, Coats AJS, Tsutsui H, Abdelhamid CM, Adamopoulos S, Albert N, et al Universal definition and classification of heart failure: a report of the Heart Failure Society of America, Heart Failure Association of the European Society of Cardiology, Japanese Heart Failure Society and Writing Committee of the Universal Definition of Heart Failure: Endorsed by the Canadian Heart Failure Society, Heart Failure Association of India, Cardiac Society of Australia and New Zealand, and Chinese Heart Failure Association. Eur J Heart Fail 2021;23:352–80. 10.1002/ejhf.2115 33605000

[4] McMurray JJ, Packer M, Desai AS, Gong J, Lefkowitz MP, Rizkala AR, et al Angiotensin-neprilysin inhibition versus enalapril in heart failure. N Engl J Med 2014;371:993–1004. 10.1056/NEJMoa1409077 25176015

[5] Lewis EF, Claggett BL, McMurray JJV, Packer M, Lefkowitz MP, Rouleau JL, et al Health-related quality of life outcomes in PARADIGM-HF. Circ Heart Fail 2017;10:e003430. 10.1161/CIRCHEARTFAILURE.116.003430 28784687

[6] United States Food and Drug Administration . https://www.accessdata.fda.gov/scripts/cder/daf/index.cfm?event=overview.process&varApplNo=207620 (12 June 2024, date last accessed).

[7] European Medicines Agency . https://www.ema.europa.eu/en/medicines/human/EPAR/entresto (12 June 2024, date last accessed).

[8] Ponikowski P, Voors AA, Anker SD, Bueno H, Cleland JGF, Coats AJS, et al 2016 ESC Guidelines for the diagnosis and treatment of acute and chronic heart failure: the Task Force for the diagnosis and treatment of acute and chronic heart failure of the European Society of Cardiology (ESC)Developed with the special contribution of the Heart Failure Association (HFA) of the ESC. Eur Heart J 2016;37:2129–200. 10.1093/eurheartj/ehw128 27206819

[9] WRITING COMMITTEE MEMBERS; Yancy CW, Jessup M, Bozkurt B, Butler J, Casey DE, et al 2016 ACC/AHA/HFSA Focused Update on New Pharmacological Therapy for Heart Failure: An Update of the 2013 ACCF/AHA Guideline for the Management of Heart Failure: A Report of the American College of Cardiology/American Heart Association Task Force on Clinical Practice Guidelines and the Heart Failure Society of America. Circulation 2016;134:e282–93. 10.1161/CIR.0000000000000435 27208050

[10] Velazquez EJ, Morrow DA, DeVore AD, Duffy CI, Ambrosy AP, McCague K, et al Angiotensin-neprilysin inhibition in acute decompensated heart failure. N Engl J Med 2019;380:539–48. 10.1056/NEJMoa1812851 30415601

[11] Wachter R, Senni M, Belohlavek J, Straburzynska-Migaj E, Witte KK, Kobalava Z, et al Initiation of sacubitril/valsartan in haemodynamically stabilised heart failure patients in hospital or early after discharge: primary results of the randomised TRANSITION study. Eur J Heart Fail 2019;21:998–1007. 10.1002/ejhf.1498 31134724

[12] Lim YMF, Molnar M, Vaartjes I, Savarese G, Eijkemans MJC, Uijl A, et al Generalizability of randomized controlled trials in heart failure with reduced ejection fraction. Eur Heart J Qual Care Clin Outcomes 2022;8:761–9. 10.1093/ehjqcco/qcab070 34596659 PMC9603541

[13] Page MJ, McKenzie JE, Bossuyt PM, Boutron I, Hoffmann TC, Mulrow CD, et al The PRISMA 2020 statement: an updated guideline for reporting systematic reviews. BMJ 2021;372:n71. 10.1136/bmj.n71 33782057 PMC8005924

[14] Wells GA, Shea B, O’Connell D, Peterson J, Welch V, Losos M, et al The Newcastle-Ottawa Scale (NOS) for assessing the quality of nonrandomised studies in meta-analyses. URL Ottawa Hospital Research Institute. http://www.ohri.ca/programs/clinical_epidemiology/oxford.htm (18 August 2025, date last accessed).

[15] Aimo A, Gaggin HK, Barison A, Emdin M, Januzzi JL Jr. Imaging, biomarker, and clinical predictors of cardiac remodeling in heart failure with reduced ejection fraction. JACC Heart Fail 2019;7:782–94. 10.1016/j.jchf.2019.06.004 31401101

[16] Daubert MA, Adams K, Yow E, Barnhart HX, Douglas PS, Rimmer S, et al NT-proBNP goal achievement is associated with significant reverse remodeling and improved clinical outcomes in HFrEF. JACC Heart Fail 2019;7:158–68. 10.1016/j.jchf.2018.10.014 30611722

[17] Goliasch G, Bartko PE, Pavo N, Neuhold S, Wurm R, Mascherbauer J, et al Refining the prognostic impact of functional mitral regurgitation in chronic heart failure. Eur Heart J 2018;39:39–46. 10.1093/eurheartj/ehx402 29020337

[18] Desai AS, Solomon SD, Shah AM, Claggett BL, Fang JC, Izzo J, et al Effect of sacubitril-valsartan vs enalapril on aortic stiffness in patients with heart failure and reduced ejection fraction: a randomized clinical trial. JAMA 2019;322:1077–84. 10.1001/jama.2019.12843 31475296 PMC6749534

[19] Kang DH, Park SJ, Shin SH, Hong G-R, Lee S, Kim M-S, et al Angiotensin receptor neprilysin inhibitor for functional mitral regurgitation. Circulation 2019;139:1354–65. 10.1161/CIRCULATIONAHA.118.037077 30586756

[20] Januzzi JL Jr, Prescott MF, Butler J, Felker GM, Maisel AS, McCague K, et al Association of change in n-terminal pro-B-type natriuretic peptide following initiation of sacubitril-valsartan treatment with cardiac structure and function in patients with heart failure with reduced ejection fraction. JAMA 2019;322:1085–95. 10.1001/jama.2019.12821 31475295 PMC6724151

[21] Januzzi JL Jr, Camacho A, Piña IL, Rocha R, Williamson KM, Maisel AS, et al Reverse cardiac remodeling and outcome after initiation of sacubitril/valsartan. Circ Heart Fail 2020;13:e006946. 10.1161/CIRCHEARTFAILURE.119.006946 32482089

[22] Januzzi JL, Omar AMS, Liu Y, Murphy S, Butler J, Felker GM, et al Association between sacubitril/valsartan initiation and mitral regurgitation severity in heart failure with reduced ejection fraction: the PROVE-HF study. Circulation 2022;146:1638–40. 10.1161/CIRCULATIONAHA.122.061693 36183276 PMC9674443

[23] Mohebi R, Liu Y, Piña IL, Prescott MF, Butler J, Felker GM, et al Dose-response to sacubitril/valsartan in patients with heart failure and reduced ejection fraction. J Am Coll Cardiol 2022;80:1529–41. 10.1016/j.jacc.2022.08.737 36229089

[24] Chang PC, Wang CL, Hsiao FC, Wen M-S, Huang C-Y, Chou C-C, et al Sacubitril/valsartan vs. angiotensin receptor inhibition in heart failure: a real-world study in Taiwan. ESC Heart Fail 2020;7:3003–12. 10.1002/ehf2.12924 32720478 PMC7524065

[25] Chang HY, Chen KC, Fong MC, Feng A-N, Fu H-N, Huang K-C, et al Recovery of left ventricular dysfunction after sacubitril/valsartan: predictors and management. J Cardiol 2020;75:233–41. 10.1016/j.jjcc.2019.08.005 31563433

[26] Park JJ, Lee SE, Cho HJ, Choi J-O, Yoo B-S, Kang S-M, et al Real-world usage of sacubitril/valsartan in Korea: a multi-center, retrospective study. Int J Heart Fail 2022;4:193–204. 10.36628/ijhf.2022.0015 36381016 PMC9634027

[27] Ekici B, Yaman M, Küçük M, Dereli S, Yenercag M, Yigit Z, et al Angiotensin receptor neprilysin inhibitor for patients with heart failure and reduced ejection fraction: real-world experience from Turkey (ARNi-TR). Turk Kardiyol Dern Ars 2021;49:357–67. 10.5543/tkda.2021.63099 34308869

[28] Di Lenarda A, Di Gesaro G, Sarullo FM, Miani D, Driussi M, Correale M, et al Sacubitril/valsartan in heart failure with reduced ejection fraction: real-world experience from Italy (the REAL.IT Study). J Clin Med 2023;12:699. 10.3390/jcm12020699 36675628 PMC9863394

[29] Mapelli M, Mattavelli I, Paolillo S, Salvioni E, Magrì D, Galotta A, et al Effects of sacubitril/valsartan on exercise capacity: a prognostic improvement that starts during uptitration. Eur J Clin Pharmacol 2023;79:1173–84. 10.1007/s00228-023-03527-y 37368004 PMC10427709

[30] Giallauria F, Vitale G, Pacileo M, Di Lorenzo A, Oliviero A, Passaro F, et al Sacubitril/valsartan improves autonomic function and cardiopulmonary parameters in patients with heart failure with reduced ejection fraction. J Clin Med 2020;9:1897. 10.3390/jcm9061897 32560431 PMC7356720

[31] Campanile A, Visco V, De Carlo S, Ferruzzi GJ, Mancusi C, Izzo C, et al Sacubitril/valsartan vs. standard medical therapy on exercise capacity in HFrEF patients. Life (Basel) 2023;13:1174. 10.3390/life13051174 37240819 PMC10220971

[32] Heidenreich PA, Bozkurt B, Aguilar D, Allen LA, Byun JJ, Colvin MM, et al 2022 AHA/ACC/HFSA guideline for the management of heart failure: a report of the American College of Cardiology/American Heart Association Joint Committee on Clinical Practice Guidelines. Circulation 2022;145:e895–1032. 10.1161/CIR.0000000000001063 35363499

[33] Authors/Task Force Members; McDonagh TA, Metra M, Adamo M, Gardner RS, Baumbach A, et al 2021 ESC Guidelines for the diagnosis and treatment of acute and chronic heart failure: developed by the Task Force for the diagnosis and treatment of acute and chronic heart failure of the European Society of Cardiology (ESC). With the special contribution of the Heart Failure Association (HFA) of the ESC. Eur J Heart Fail 2022;24:4–131. 10.1002/ejhf.2333 35083827

[34] Felker GM, Butler J, Ibrahim NE, Piña IL, Maisel A, Bapat D, et al Implantable cardioverter-defibrillator eligibility after initiation of sacubitril/valsartan in chronic heart failure: insights from PROVE-HF. Circulation 2021;144:180–2. 10.1161/CIRCULATIONAHA.121.054034 34251893 PMC8270225

[35] Pastore MC, Mandoli GE, Giannoni A, Benfari G, Dini FL, Pugliese NR, et al Sacubitril/valsartan reduces indications for arrhythmic primary prevention in heart failure with reduced ejection fraction: insights from DISCOVER-ARNI, a multicenter Italian register. Eur Heart J Open 2021;2:oeab046. 10.1093/ehjopen/oeab046 35919657 PMC9242049

[36] Monzo L, Gaudio C, Cicogna F, Tota C, Petronilli V, Mennuni S, et al Impact of sacubitril/valsartan on implantable defibrillator eligibility in heart failure: a real-world experience. Eur Rev Med Pharmacol Sci 2021;25:5690–700. 10.26355/eurrev_202109_26788 34604961

[37] Nogueira MA, Brochado M, Nabais I, Batista É, Matias C, Proença G. Is Sacubitril/valsartan able to change the timing for implantation of cardiac devices in heart failure with reduced ejection fraction? Hearts 2022;3:88–95. 10.3390/hearts3030012

[38] Heo S, Lennie TA, Okoli C, Moser DK. Quality of life in patients with heart failure: ask the patients. Heart Lung 2009;38:100–8. 10.1016/j.hrtlng.2008.04.002 19254628 PMC2671196

[39] Zambroski CH, Moser DK, Bhat G, Ziegler C. Impact of symptom prevalence and symptom burden on quality of life in patients with heart failure. Eur J Cardiovasc Nurs 2005;4:198–206. 10.1016/j.ejcnurse.2005.03.010 15916924

[40] Konstam V, Salem D, Pouleur H, Kostis J, Gorkin L, Shumaker S, et al Baseline quality of life as a predictor of mortality and hospitalization in 5,025 patients with congestive heart failure. SOLVD Investigations. Studies of Left Ventricular Dysfunction Investigators. Am J Cardiol 1996;78:890–5. 10.1016/s0002-9149(96)00463-8 8888661

[41] Chandra A, Lewis EF, Claggett BL, Desai AS, Packer M, Zile MR, et al Effects of sacubitril/valsartan on physical and social activity limitations in patients with heart failure: a secondary analysis of the PARADIGM-HF trial. JAMA Cardiol 2018;3:498–505. 10.1001/jamacardio.2018.0398 29617523 PMC6128510

[42] Khariton Y, Fonarow GC, Arnold SV, Hellkamp A, Nassif ME, Sharma PP, et al Association between sacubitril/valsartan initiation and health status outcomes in heart failure with reduced ejection fraction. JACC Heart Fail 2019;7:933–41. 10.1016/j.jchf.2019.05.016 31521679 PMC7122134

[43] Thomas M, Khariton Y, Fonarow GC, Arnold SV, Hill L, Nassif ME, et al Association between sacubitril/valsartan initiation and real-world health status trajectories over 18 months in heart failure with reduced ejection fraction. ESC Heart Fail 2021;8:2670–8. 10.1002/ehf2.13298 33932120 PMC8318450

[44] Chapman B, Hellkamp AS, Thomas LE, Albert NM, Butler J, Patterson JH, et al Angiotensin receptor neprilysin inhibition and associated outcomes by race and ethnicity in patients with heart failure with reduced ejection fraction: data from CHAMP-HF. J Am Heart Assoc. 2022;11:e022889. 10.1161/JAHA.121.022889 35722989 PMC9238653

[45] Piepoli MF, Spoletini I, Rosano G. Monitoring functional capacity in heart failure. Eur Heart J Suppl 2019;21:M9–M12. 10.1093/eurheartj/suz216 31908608 PMC6937504

[46] Piepoli MF, Hussain RI, Comin-Colet J, Dosantos R, Ferber P, Jaarsma T, et al OUTSTEP-HF: randomised controlled trial comparing short-term effects of sacubitril/valsartan versus enalapril on daily physical activity in patients with chronic heart failure with reduced ejection fraction. Eur J Heart Fail 2021;23:127–35. 10.1002/ejhf.2076 33314487

[47] Beltrán P, Palau P, Domínguez E, Faraudo M, Núñez E, Guri O, et al Sacubitril/valsartan and short-term changes in the 6-minute walk test: a pilot study. Int J Cardiol 2018;252:136–9. 10.1016/j.ijcard.2017.10.074 29249422

[48] Vitale G, Romano G, Di Franco A, Caccamo G, Nugara C, Ajello L, et al Early effects of sacubitril/valsartan on exercise tolerance in patients with heart failure with reduced ejection fraction. J Clin Med 2019;8:262. 10.3390/jcm8020262 30791533 PMC6406731

[49] Nugara C, Giallauria F, Vitale G, Sarullo S, Gentile G, Clemenza F, et al Effects of sacubitril/valsartan on exercise capacity in patients with heart failure with reduced ejection fraction and the role of percentage of delayed enhancement measured by cardiac magnetic resonance in predicting therapeutic response: a multicentre study. Card Fail Rev 2023;9:e07. 10.15420/cfr.2022.13 37427008 PMC10326660

[50] Proudfoot C, Studer R, Rajput T, Jindal R, Agrawal R, Corda S, et al Real-world effectiveness and safety of sacubitril/valsartan in heart failure: a systematic review. Int J Cardiol 2021;331:164–71. 10.1016/j.ijcard.2021.01.061 33545266

[51] Abdin A, Schulz M, Riemer U, Hadëri B, Wachter R, Laufs U, et al Sacubitril/valsartan in heart failure: efficacy and safety in and outside clinical trials. ESC Heart Fail 2022;9:3737–50. 10.1002/ehf2.14097 35921043 PMC9773772

[52] Rahhal A, Kasem M, Orabi B, Hamou F, Abuyousef S, Mahfouz A, et al Effectiveness of sacubitril/valsartan in heart failure with reduced ejection fraction using real-world data: an updated systematic review and meta-analysis. Curr Probl Cardiol 2023;48:101412. 10.1016/j.cpcardiol.2022.101412 36170910

[53] Mohanty AF, Levitan EB, Dodson JA, Vardeny O, King JB, LaFleur J, et al Characteristics and healthcare utilization among veterans treated for heart failure with reduced ejection fraction who switched to sacubitril/valsartan. Circ Heart Fail 2019;12:e005691. 10.1161/CIRCHEARTFAILURE.118.005691 31718321

[54] Ozaki AF, Krumholz HM, Mody FV, Jackevicius CA. National trends in the use of sacubitril/valsartan. J Card Fail 2021;27:839–47. 10.1016/j.cardfail.2021.05.015 34364661

[55] Sumarsono A, Vaduganathan M, Ajufo E, Navar AM, Fonarow GC, Das SR, et al Contemporary patterns of medicare and medicaid utilization and associated spending on sacubitril/valsartan and ivabradine in heart failure. JAMA Cardiol 2020;5:336–9. 10.1001/jamacardio.2019.4982 31738371 PMC6865331

[56] Stolfo D, Benson L, Lindberg F, Dahlström U, Käck O, Sinagra G, et al Status and timing of angiotensin receptor-neprilysin inhibitor implementation in patients with heart failure and reduced ejection fraction: data from the Swedish Heart Failure Registry. Eur J Heart Fail 2024;26:2243–57. 10.1002/ejhf.3404 39078343

[57] Savarese G, Hage C, Benson L, Schrage B, Thorvaldsen T, Lundberg A, et al Eligibility for sacubitril/valsartan in heart failure across the ejection fraction spectrum: real-world data from the Swedish Heart Failure Registry. J Intern Med 2021;289:369–84. 10.1111/joim.13165 32776357 PMC7984286

[58] D'Amario D, Rodolico D, Rosano GMC, Dahlström U, Crea F, Lund LH, et al Association between dosing and combination use of medications and outcomes in heart failure with reduced ejection fraction: data from the Swedish Heart Failure Registry. Eur J Heart Fail 2022;24:871–84. 10.1002/ejhf.2477 35257446 PMC9315143

[59] Wachter R, Fonseca AF, Balas B, Kap E, Engelhard J, Schlienger R, et al Real-world treatment patterns of sacubitril/valsartan: a longitudinal cohort study in Germany. Eur J Heart Fail 2019;21:588–97. 10.1002/ejhf.1465 30972918 PMC6607491

[60] Peri-Okonny PA, Mi X, Khariton Y, Patel KK, Thomas L, Fonarow GC, et al Target doses of heart failure medical therapy and blood pressure: insights from the CHAMP-HF registry. JACC Heart Fail 2019;7:350–8. 10.1016/j.jchf.2018.11.011 30738978 PMC6440823

[61] Berg DD, Braunwald E, DeVore AD, Lala A, Pinney SP, Duffy CI, et al Efficacy and safety of sacubitril/valsartan by dose level achieved in the PIONEER-HF trial. JACC Heart Fail 2020;8:834–43. 10.1016/j.jchf.2020.06.008 32800511 PMC7541586

[62] Vardeny O, Claggett B, Packer M, Zile MR, Rouleau J, Swedberg K, et al Efficacy of sacubitril/valsartan vs. enalapril at lower than target doses in heart failure with reduced ejection fraction: the PARADIGM-HF trial. Eur J Heart Fail 2016;18:1228–34. 10.1002/ejhf.580 27283779 PMC5095784

[63] Tomasoni D, Adamo M, Bozkurt B, Heidenreich P, McDonagh T, Rosano GMC, et al Aiming at harmony. Comparing and contrasting international HFrEF Guidelines. Eur Heart J Suppl 2022;24:L20–8. 10.1093/eurheartjsupp/suac124 36545230 PMC9762876

[64] Fu M, Vedin O, Svennblad B, Lampa E, Johansson D, Dahlström U, et al Implementation of sacubitril/valsartan in Sweden: clinical characteristics, titration patterns, and determinants. ESC Heart Fail 2020;7:3633–43. 10.1002/ehf2.12883 32881399 PMC7754959

[65] Badreldin HA, Korayem GB, Alenazy BA, Aljohani MH, Alshaya OA, Al Sulaiman K, et al Real-world analysis of integration of sacubitril/valsartan into clinical practice in Saudi Arabia. Medicine (Baltimore) 2023;102:e36699. 10.1097/MD.0000000000036699 38134075 PMC10735148

[66] Mohanty AF, Levitan EB, King JB, Dodson JA, Vardeny O, Cook J, et al Sacubitril/valsartan initiation among veterans who are renin-angiotensin-aldosterone system inhibitor naïve with heart failure and reduced ejection fraction. J Am Heart Assoc 2021;10:e020474. 10.1161/JAHA.120.020474 34612065 PMC8751890

[67] Carnicelli AP, Lippmann SJ, Greene SJ, Mentz RJ, Greiner MA, Hardy NC, et al Sacubitril/valsartan initiation and postdischarge adherence among patients hospitalized for heart failure. J Card Fail 2021;27:826–36. 10.1016/j.cardfail.2021.03.012 34364659

[68] Savarese G, Bodegard J, Norhammar A, Sartipy P, Thuresson M, Cowie MR, et al Heart failure drug titration, discontinuation, mortality and heart failure hospitalization risk: a multinational observational study (US, UK and Sweden). Eur J Heart Fail 2021;23:1499–511. 10.1002/ejhf.2271 34132001

[69] López-Azor JC, Vicent L, Valero-Masa MJ, Esteban-Fernández A, Gómez-Bueno M, Pérez Á, et al Safety of sacubitril/valsartan initiated during hospitalization: data from a non-selected cohort. ESC Heart Fail 2019;6:1161–6. 10.1002/ehf2.12527 31701680 PMC6989298

[70] Savarese G, Kishi T, Vardeny O, Adamsson Eryd S, Bodegård J, Lund LH, et al Heart failure drug treatment-inertia, titration, and discontinuation: a multinational observational study (EVOLUTION HF). JACC Heart Fail 2023;11:1–14. 10.1016/j.jchf.2022.08.009 36202739

[71] Houchen E, Loefroth E, Schlienger R, Proudfoot C, Corda S, Saha S, et al Hospitalization rates in patients with heart failure and reduced ejection fraction initiating sacubitril/valsartan or angiotensin-converting enzyme inhibitors/angiotensin receptor blockers: a retrospective cohort study. Cardiol Ther 2022;11:113–27. 10.1007/s40119-021-00252-4 35094306 PMC8800553

[72] Eworuke E, Welch EC, Haug N, Horgan C, Lee HS, Zhao Y, et al Comparative risk of angioedema with sacubitril-valsartan vs renin-angiotensin-aldosterone inhibitors. J Am Coll Cardiol 2023;81:321–31. 10.1016/j.jacc.2022.10.033 36697132

[73] Bozkurt B, Nair AP, Misra A, Scott CZ, Mahar JH, Fedson S. Neprilysin inhibitors in heart failure: the science, mechanism of action, clinical studies, and unanswered questions. JACC Basic Transl Sci 2022;8:88–105. 10.1016/j.jacbts.2022.05.010 36777165 PMC9911324

[74] Savarese G, Lindberg F, Cannata A, Chioncel O, Stolfo D, Musella F, et al How to tackle therapeutic inertia in heart failure with reduced ejection fraction. A scientific statement of the Heart Failure Association of the ESC. Eur J Heart Fail 2024;26:1278–97. 10.1002/ejhf.3295 38778738

[75] Savarese G, Lindberg F, Christodorescu RM, Ferrini M, Kumler T, Toutoutzas K, et al Physician perceptions, attitudes, and strategies towards implementing guideline-directed medical therapy in heart failure with reduced ejection fraction. A survey of the Heart Failure Association of the ESC and the ESC Council for Cardiology Practice. Eur J Heart Fail 2024;26:1408–18. 10.1002/ejhf.3214 38515385

[76] Sangaralingham LR, Sangaralingham SJ, Shah ND, Yao X, Dunlay SM. Adoption of sacubitril/valsartan for the management of patients with heart failure. Circ Heart Fail 2018;11:e004302. 10.1161/CIRCHEARTFAILURE.117.004302 29453287 PMC5820776

[77] Tan NY, Sangaralingham LR, Sangaralingham SJ, Yao X, Shah ND, Dunlay SM. Comparative effectiveness of sacubitril-valsartan versus ACE/ARB therapy in heart failure with reduced ejection fraction. JACC Heart Fail 2020;8:43–54. 10.1016/j.jchf.2019.08.003 31838035 PMC8356205

[78] Carnicelli AP, Li Z, Greiner MA, Lippmann SJ, Greene SJ, Mentz RJ, et al Sacubitril/valsartan adherence and postdischarge outcomes among patients hospitalized for heart failure with reduced ejection fraction. JACC Heart Fail 2021;9:876–86. 10.1016/j.jchf.2021.06.018 34509408

[79] Cho DH, Choi J, Youn JC, Kim M-N, Lee CJ, Son J-W, et al Angiotensin receptor-neprilysin inhibitor adherence and outcomes in heart failure with reduced ejection fraction. ESC Heart Fail 2025;12:603–12. 10.1002/ehf2.15117 39420468 PMC11769608

[80] Vardeny O, Claggett B, Kachadourian J, Pearson SM, Desai AS, Packer M, et al Incidence, predictors, and outcomes associated with hypotensive episodes among heart failure patients receiving sacubitril/valsartan or enalapril: the PARADIGM-HF trial (prospective comparison of angiotensin receptor neprilysin inhibitor with angiotensin-converting enzyme inhibitor to determine impact on global mortality and morbidity in heart failure). Circ Heart Fail 2018;11:e004745. 10.1161/CIRCHEARTFAILURE.117.004745 29643067

[81] Damman K, Gori M, Claggett B, Jhund PS, Senni M, Lefkowitz MP, et al Renal effects and associated outcomes during angiotensin-neprilysin inhibition in heart failure. JACC Heart Fail 2018;6:489–98. 10.1016/j.jchf.2018.02.004 29655829

[82] Desai AS, Vardeny O, Claggett B, McMurray JJV, Packer M, Swedberg K, et al Reduced risk of hyperkalemia during treatment of heart failure with mineralocorticoid receptor antagonists by use of sacubitril/valsartan compared with enalapril: a secondary analysis of the PARADIGM-HF trial. JAMA Cardiol 2017;2:79–85. 10.1001/jamacardio.2016.4733 27842179

[83] Bhatt AS, Vaduganathan M, Claggett BL, Liu J, Packer M, Desai AS, et al Effect of sacubitril/valsartan vs. enalapril on changes in heart failure therapies over time: the PARADIGM-HF trial. Eur J Heart Fail 2021;23:1518–24. 10.1002/ejhf.2259 34101308 PMC9291580

[84] Kim YS, Brar S, D'Albo N, Dey A, Shah S, Ganatra S, et al Five years of sacubitril/valsartan-a safety analysis of randomized clinical trials and real-world pharmacovigilance. Cardiovasc Drugs Ther 2022;36:915–24. 10.1007/s10557-021-07210-1 34125356

[85] Mann DL, Givertz MM, Vader JM, Starling RC, Shah P, McNulty SE, et al Effect of treatment with sacubitril/valsartan in patients with advanced heart failure and reduced ejection fraction: a randomized clinical trial. JAMA Cardiol 2022;7:17–25. 10.1001/jamacardio.2021.4567 34730769 PMC8567189

[86] Vader JM, Givertz MM, Starling RC, McNulty SE, Anstrom KJ, Desvigne-Nickens P, et al Tolerability of sacubitril/valsartan in patients with advanced heart failure: analysis of the LIFE trial run-in. JACC Heart Fail 2022;10:449–56. 10.1016/j.jchf.2022.04.013 35772853

[87] Desai AS, Solomon S, Claggett B, McMurray JJV, Rouleau J, Swedberg K, et al Factors associated with noncompletion during the run-in period before randomization and influence on the estimated benefit of LCZ696 in the PARADIGM-HF trial. Circ Heart Fail 2016;9:e002735. 10.1161/CIRCHEARTFAILURE.115.002735 27296397

[88] Skouri H, Girerd N, Monzo L, Petrie MC, Böhm M, Adamo M, et al Clinical management and therapeutic optimization of patients with heart failure with reduced ejection fraction and low blood pressure. A clinical consensus statement of the Heart Failure Association (HFA) of the ESC. Eur J Heart Fail 2025;27:707–22. 10.1002/ejhf.3618 40012353

[89] Kaddoura R, Abushanab D, Arabi AR, Alyafei SA, Al-Badriyeh D. Cost-effectiveness analysis of sacubitril/valsartan for reducing the use of implantable cardioverter-defibrillator (ICD) and the risk of death in ICD-eligible heart failure patients with reduced ejection fraction. Curr Probl Cardiol 2022;47:101385. 10.1016/j.cpcardiol.2022.101385 36063914

[90] Dixit NM, Parikh NU, Ziaeian B, Jackson N, Fonarow GC. Cost-effectiveness of comprehensive quadruple therapy for heart failure with reduced ejection fraction. JACC Heart Fail 2023;11:541–51. 10.1016/j.jchf.2023.01.004 36892492

[91] Garcia-Quintana A, Javier Parrondo J. Cost-effectiveness of quadruple therapy for heart failure with reduced ejection fraction (HFrEF) in Spain. Presented at HFA Heart Failure Congress 2024. https://esc365.escardio.org/presentation/280437 (12 January 2025, date last accessed).

[92] Fonarow GC, Hernandez AF, Solomon SD, Yancy CW. Potential mortality reduction with optimal implementation of angiotensin receptor neprilysin inhibitor therapy in heart failure. JAMA Cardiol 2016;1:714–7. 10.1001/jamacardio.2016.1724 27437874

[93] Tromp J, Ouwerkerk W, van Veldhuisen DJ, Hillege HL, Richards AM, van der Meer P, et al A systematic review and network meta-analysis of pharmacological treatment of heart failure with reduced ejection fraction. JACC Heart Fail 2022;10:73–84. 10.1016/j.jchf.2021.09.004 34895860

[94] Nguyen AH, Hurwitz M, Abraham J, Blumer V, Flanagan MC, Garan AR, et al Medical management and device-based therapies in chronic heart failure. J Soc Cardiovasc Angiogr Interv 2023;2:101206. 10.1016/j.jscai.2023.101206 39131076 PMC11308856

