# Supplementary material for: Ten years real-world experience with sacubitril/valsartan in patients with heart failure with reduced ejection fraction
Source: ESC Heart Fail. 2026 Mar 31;13(2):xvag095. doi: 10.1093/eschf/xvag095 (PMC13122610; doi:10.1093/eschf/xvag095)
Supplement: xvag095_Supplementary_Data [file xvag095_supplementary_data.docx]

**SUPPLEMENTARY APPENDIX**

**Literature search strategy**

*Preliminary search string*

((sacu*[Title/Abstract]) OR (nepr*[Title/Abstract]) OR (arni[Title/Abstract]) OR (entresto[Title/Abstract] OR (LCZ696[Title/Abstract])) AND ((obse*[Title/Abstract]) OR (regi*[Title/Abstract]) OR (real*[Title/Abstract]) OR (prac*[Title/Abstract]) OR (retr*[Title/Abstract]) OR (pros*[Title/Abstract]) OR (stud*[Title/Abstract])) NOT (random*[Title])) AND (("2016/01/01"[Date - Publication] : "3000"[Date - Publication]))

Truncation of words was performed by introducing asterisks to include all the words with these letters, as reported in the section titled “Truncating search terms” in the PubMed research guide available at <https://pubmed.ncbi.nlm.nih.gov/help/#narrow-search>

*Final search string*

[((sacu*[Title/Abstract]) OR (nepr*[Title/Abstract]) OR (arni[Title/Abstract]) OR (entresto[Title/Abstract] OR (LCZ696[Title/Abstract])) AND ((obse*[Title/Abstract]) OR (regi*[Title/Abstract]) OR (real*[Title/Abstract]) OR (prac*[Title/Abstract]) OR ( - Search Results - PubMed (nih.gov)](https://urldefense.com/v3/__https:/pubmed.ncbi.nlm.nih.gov/?term=((sacu**A5BTitle*2FAbstract*5D)*20OR*20(nepr**A5BTitle*2FAbstract*5D)*20OR*20(arni*5BTitle*2FAbstract*5D)*20OR*20(entresto*5BTitle*2FAbstract*5D*20OR*20(LCZ696*5BTitle*2FAbstract*5D))*20AND*20((obse**A5BTitle*2FAbstract*5D)*20OR*20(regi**A5BTitle*2FAbstract*5D)*20OR*20(real**A5BTitle*2FAbstract*5D)*20OR*20(prac**A5BTitle*2FAbstract*5D)*20OR*20(retr**A5BTitle*2FAbstract*5D)*20OR*20(pros**A5BTitle*2FAbstract*5D)*20OR*20(stud**A5BTitle*2FAbstract*5D))*20NOT*20(random**A5BTitle*5D))*20AND*20((*222016*2F01*2F01*22*5BDate*20-*20Publication*5D*20*3A*20*223000*22*5BDate*20-*20Publication*5D))&filter=simsearch3.fft&filter=pubt.clinicaltrialphaseiv&filter=pubt.consensusdevelopmentconference&filter=pubt.consensusdevelopmentconferencenih&filter=pubt.guideline&filter=pubt.meta-analysis&filter=pubt.observationalstudy&filter=pubt.practiceguideline&filter=pubt.review&filter=pubt.scientificintegrityreview&filter=pubt.systematicreview&filter=lang.english&show_snippets=off&format=abstract&size=200__;KiUlJSUlKiUlJSUlJSUlJSUlJSUlJSUlJSUlKiUlJSUlKiUlJSUlKiUlJSUlKiUlJSUlKiUlJSUlKiUlJSUlKiUlJSUlKiUlJSUlJSUlJSUlJSUlJSUlJSUlJQ!!N3hqHg43uw!p3NnZD3Bcw3fqF-6nar579u34y8dxA07yIGJHeEbkhlW4Y0wgdI9R4U4eZ6HTvc42E09HeWBv4xh4gylyYjgWw1oDPYA8Q$)

The final search string was derived using filters for English language, full-text articles, clinical trial, phase IV, consensus development conference, consensus development conference, NIH, guideline, meta-analysis, observational study, practice guideline, review, scientific integrity review, and systematic review.

We also used a nontruncated search string to retrieve any additional articles:

(((((((((((((heart failure) OR (myocardial failure)) OR (HFpEF)) OR (HFrEF)) OR (hypertension)) OR (heart decompensation)) OR (cardiac failure)) AND (sacubitril/valsartan)) OR (sacubitril-valsartan)) OR (Sac/Val)) OR (lcz696)) OR (entresto)) OR (neprilysin inhibitor)) OR (ARNI)

**Study selection**

The main search string was developed and provided by the authors, while the non-truncated search string was developed by the supporting medical writers. The subsequent literature search, title and abstract screening, and data extraction were performed independently and in a double-blind fashion by both the author team and the supporting medical writers. Inclusion criteria consisted of publications in english language on retrospective and prospective observational studies and registries with a focus on Sac/Val use in HFrEF. Study selection was independently performed by two reviewers, with discrepancies resolved through discussion. The quality and risk of bias of the included studies were assessed using the Newcastle Ottawa Scale (NOS) for cohort studies.

**Quality assessment**
The following parameters were checked to evaluate the quality of the included studies: selection (representativeness of the intervention cohort, selection of the nonintervention cohort, ascertainment of intervention, outcome of interest not present at start of study), comparability (of cohorts on the basis of the design or analysis), and outcomes (assessment of outcome, follow-up long enough for the outcomes to occur, adequacy of follow-up of cohorts).

**Supplementary Table 1.** Key Characteristics of the Included Studies

| **Source and country** | **Design** | **Patients treated with sacubitril/valsartan** | **Age**  **(years)** | **Female (%)** | **Follow-up (months)** | **Main outcome assessed** | **Summary of Key Findings** |
| --- | --- | --- | --- | --- | --- | --- | --- |
| **Source:** PROVE-HF^1-5^  **Country:** US | Prospective, single-group, open-label study in 78 outpatient sites | 794 | 65.1 (12.4) | 28.5 | 12 | NT-proBNP correlation with reverse remodeling^1^ | Reduction in NT-proBNP concentration was significantly correlated with improvements in cardiac volume and function |
|  |  |  |  |  |  | Association of reverse remodeling achievement with HHF and mortality^2^ | The odds of HHF or death were significantly higher for patients with below-average linear slopes for NT-proBNP and LVEDVi compared with patients with above-average linear slopes for both measures (OR: 2.03; 95% CI: 1.25–3.30) |
|  |  |  |  |  |  | Association between Sac/Val and change in MR severity^3^ | Prevalence of 3 to 4+ MR decreased to 8.4% (relative 44.7% of reduction) |
|  |  |  |  |  |  | Associations between Sac/Val doses and changes in prognostic biomarkers, health status, and cardiac remodeling^4^ | Similar changes in KCCQ-23 score, NT-proBNP, left ventricular and atrial volumes, and EF between different doses of Sac/Val |
|  |  | 661 | 65 (NA) | 27 |  | Eligibility for ICD at 6 and 12 months in patients ICD-eligible at baseline^5^ | 32% of patients no longer eligible at 6 months, 62% at 12 months |
| **Source:** Chang Gung Research Database^6^  **Country:** Taiwan | Prospective study using retrospective collected data from electronic medical records | 502 | 62.1 (15.4) | 23.7 | 12 | Compare the effectiveness of Sac/Val vs ARB on CV death/HHF^6^ | HR: 0.74; 95% CI: 0.57–0.96 |
| **Source:** Cheng Hsin General Hospital^7^  **Country:** Taiwan | Retrospective study of patients treated with Sac/Val | 437 | 61.2 (14.5) | 25.4 | 12 | Association between Sac/Val and reverse remodeling^7^ | Nonischemic etiology of HF, smaller baseline LVEDD, and higher initial dosage of Sac/Val could predict better recovery of EF |
| **Source:** REASSURE^8^  **Country:** Korea | Retrospective multicenter study of patients treated with Sac/Val | 600 | 69.9 (13.7) | 25.8 | 12 | Evaluate treatment patterns^8^ | Most patients (95%) started with a nontarget dose of Sac/Val, with 30% achieving the target dose by the end of follow-up |
| **Source:** ARNi-TR^9^  **Country:** Turkey | Retrospective multicenter study of patients treated with Sac/Val | 704 | 64.7 (12.5) | 28.1 | 12 | Association between Sac/Val and reverse remodeling^9^ | Significant association with lower NT-proBNP and higher EF |
| **Source:** REAL.IT^10^  **Country:** Italy | Retrospective cohort study based on electronic medical records from 9 outpatient sites | 924 | 64.5 (11.9) | 15.4 | 12 | Association between Sac/Val and reverse remodeling^10^ | EF improved by ≥5% in 56.3% of patients; 39.7% had a ≥30% of reduction of NT-proBNP |
| **Source:** ARIADNE registry^11^  **Country:** 17 European countries | Prospective, observational registry of patients with HFrEF treated in 687 outpatient sites | 4614 | 67.3 (11.5) | 23.6 | 12 | Compare baseline characteristics of patients initiated on Sac/Val vs conventional therapy^11^ | Patients on Sac/Val were more likely to have NYHA class III/IV, lower EF, and be on triple HF therapy with a beta-blocker and MRA |
| **Source:** CHAMP-HF registry^12-16^  **Country:** US | Prospective observational cohort study of outpatients with HFrEF in 152 sites | 616 | 62.6 (13.1) | 31.5 | 12 | Predictors of Sac/Val prescription^12^ | Patients prescribed Sac/Val were younger, less likely to have CKD, more likely to have CRT and being followed in larger practices, and had lower EF |
|  |  | 508 | 64 (12.9) | 30 | 2 [1.1, 3.7] | Association of Sac/Val initiation with QoL^13^ | Greater mean improvement in KCCQ-OS in patients initiated on Sac/Val (5.3 ± 19 vs 2.5 ± 17.4, *P* < 0.001) |
|  |  | 746 | 63.4 (13.0) | 29 | 18 | Association of Sac/Val initiation with QoL^14^ | Sac/Val associated with higher KCCQ‐OSS (73.9 vs 71.3, *P* < 0.001) |
|  |  | 758 | 63.9 (13.0) | 29 | NA | Association of Sac/Val initiation with QoL across different ethnicities^15^ | Changes in KCCQ‐OSS were similar among ethnic groups |
|  |  | 400 | NA | NA | NA | Rate of the use of target doses of medical therapies^16^ | 12.9% of patients received Sac/Val, of them only 15% achieved the target dose |
| **Source:** Beltrán P et al^17^  **Country:** Spain | Prospective cohort study of patients with chronic HF from two third level centers in Spain | 58 | 70 (11) | 42 | 1 | Association of Sac/Val initiation with 6MWT distance^17^ | The 6MWT distance increased significantly at 30 days by 13.9% compared with the baseline 6MWT |
| **Source:** Mapelli M et al^18^  **Country:** Italy | Prospective cohort study of outpatients with HFrEF referred to 3 HF units for Sac/Val prescription | 96 | 63.7 (9.8) | 20 | 10.1 (2.2) | Association of Sac/Val initiation with CPET parameters^18^ | Sac/Val was associated with improvements in CPET parameters |
| **Source:** IRRB/23/15^19-21^  **Country:** Italy | Prospective cohort study of outpatients with HFrEF initiating Sac/Val | 134 | 57.9 (9.6) | 13 | 12 | Association of Sac/Val initiation with CPET parameters and heart rate recovery^19^ | Sac/Val was associated with improvements in heart rate recovery and CPET parameters |
|  |  | 99 | 58.7 (9.3) | 14 | 6.2 [3-14.9] | Association of Sac/Val initiation with CPET parameters^20^ | Sac/Val was associated with improvements in CPET parameters |
|  |  | 134 | 57.9 (9.6) | 13 | 13.3 (6.6) | Association of Sac/Val initiation with CPET parameters and CMR evaluated myocardial fibrosis^21^ | Sac/Val was associated with improvements in CPET parameters. When delayed enhancement >4.6% was detected, the association between Sac/Val and CPET parameters was significantly weaker |
| **Source:** Campanile A et al^22^  **Country:** Italy | Single-center, retrospective, cohort study of outpatients with HFrEF | 12 | 66.1 (7.9) | 16.7 | 16 [11.5-22] | Comparison of Sac/Val initiation vs optimal medical therapy on CPET parameters^22^ | There was no significant benefit of Sac/Val on CPET parameters compared with optimal medical therapy |
| **Source:** Veterans Affairs Database^23,24^  **Country:** US | Retrospective cohort study of veterans with HFrEF from 170 medical centers and 1063 outpatient sites of care | 1612 | 68.5 (10.1) | 1.6 | 4 | Comparison of characteristics of patients switching to Sac/Val vs patients treated with ACEi/ARB^23^ | Patients switching to Sac/Val were a minority (4.2%) and less likely to have a history of myocardial infarction or hypertension, and more likely to be of Black ethnicity and have a lower EF. |
|  |  | 3458 | 73.1 (10.8) | 1.1 | 6 | Describe the Sac/Val initiation rate, associated characteristics, and 6‐month follow‐up dosing among ACEi/ARB-naïve patients^24^ | Sac/Val initiators had fewer baseline CV comorbidities and the lowest proportion on ≥50% target daily dose at 6‐month follow‐up compared with ACEi/ARB initiators |
| **Source:** IQVIA National Prescription Audit Database^25^  **Country:** US | The National Prescription Audit database measures the number of prescriptions filled by retail, long-term care and mail-order pharmacies | 3.3 million prescriptions dispensed between 2016 and 2019 | NA | NA | NA | Describe Sac/Val use and dosage patterns^25^ | Over 3 years, there was a 5.6-fold increase in the number of Sac/Val prescriptions dispensed per month. In the last year only 20.6% of the dispensations were for the target dose |
| **Source:** Medicare Part D Prescription Drug Event and Medicaid Utilization and Spending dataset^26^  **Country:** US | Nationwide claims-based study | 2016 beneficiaries: 35 423  2017 beneficiaries: 90 606 | NA | NA | NA | Changes in total spending, per-beneficiary/claim spending, number of beneficiaries, and number of claims for Sac/Val^26^ | The number of Medicare beneficiaries prescribed Sac/Val increased by 156% from 2016 to 2017. The annual Medicare per-beneficiary spending on Sac/Val was $2512. Parallel trends were observed among Medicaid beneficiaries |
| **Source:** Swedish HF Registry^27-29^  **Country:** Sweden | Ongoing voluntary healthcare quality registry from 69 Swedish hospitals and primary care centers | 1065 | 69 [60-76] | 22.0 | NA | Timing, settings, and predictors of Sac/Val initiation^27^ | Sac/Val use increased from 8.3% in 2017 to 26.7% in 2021. Among hospitalized patients, 8% initiated Sac/Val in-hospital or ≤14 days after discharge. Among eligible patients with HFrEF, 8.1% of inpatients and 5.9% of outpatients initiated Sac/Val. Considering HF duration, 4.9% of patients with a HF duration of <6 months and 9.1% with a HF duration of ≥6 months initiated Sac/Val. Discontinuation at 1 year ranged between 13% and 20% across the above-reported groups |
|  |  | 21 484 | 72 [64-80] | 26.4 | NA | Eligibility to Sac/Val^28^ | In the pragmatic scenario, 67% of patients were eligible. For the literal scenario, 38% of patients were eligible. Eligible vs noneligible patients had more severe HF, more comorbidities and overall worse outcomes. |
|  |  | 1144 | 67.4 (11.2) | 20 | 19.3 | Report the introduction of Sac/Val in Sweden between April 2016 and December 2017^29^ | Patients prescribed Sac/Val were younger, more frequently male, had less prior CV disease, and lower ejection fraction. Only 9% of patients received the target dose as the initiation dose. Uptitration to the target dose was achieved in 57% of the overall cohort, with an estimated treatment persistence of 82% at 1 year. De novo use in 23% of patients. |
| **Source:** IMS longitudinal prescription database^30^  **Country:** Germany | Retrospective cohort study. The IMS database captures anonymized patient-level data on all dispensed prescriptions from retail pharmacies. | 26 191 | 71.3 (12.0) | 25.8 | 6 | Analyze the treatment pattern of Sac/Val use^30^ | Two-thirds of patients were prescribed the lowest Sac/Val dose at index and uptitration was attempted in 41% of these patients. Ten percent of patients who were prescribed the target dose at index had to be stably down-titrated. Among patients who were prescribed a nontarget dose at index that were uptitrated, > 80% remained on the higher dose |
| **Source:** Badreldin HA et al^31^  **Country:** Saudi Arabia | Retrospective cohort study of patients initiating Sac/Val | 400 | 58.6 (13.8) | 30.8 | 6 | Compare the patient characteristics with the participants of the PARADIGM-HF RCT^31^ | Compared with the PARADIGM-HF trial, the cohort had a younger mean age and a higher prevalence of diabetes mellitus. Sac/Val was initiated de novo in 34% of patients. Inpatient was the setting for treatment initiation in 90% of patients |
| **Source:** GWTG-HF Registry^32,33^  **Country:** US | Ongoing, national, prospective data collection and quality improvement initiative focused on hospitalized patients with HF | 430 | 77 [71-82] | 39.1 | 12 | Associations between timing of Sac/Val initiation and post-discharge adherence^32^ | More than 90% of eligible patients hospitalized were discharged without Sac/Val. Patients who initiated treatment as inpatients had a higher adherence than those who initiated at discharge. Only 7.7% of patients discharged without Sac/Val received it during follow-up |
|  |  | 897 | 77 [71-82] | 39.8 | 12 | Association between Sac/Val adherence and all-cause mortality and hospitalization^33^ | Compared with patients with PDC <80%, patients with PDC ≥80% had a significantly lower adjusted hazard of all-cause rehospitalization (HR: 0.69, 95% CI: 0.56-0.86) and death at 1 year (HR: 0.53, 95% CI: 0.38-0.74) |
| **Source:** Savarese G et al^34^  **Country:** US, UK, and Sweden | Electronic health records in Sweden (nationwide registries), United Kingdom (CPRD Aurum) and United States (IBM MarketScan®, Commercial and Medicare Supplemental databases) of new users of HFrEF medical therapy | 29 546 | 65 (13) | 27 | 12 | Report patterns of dose titration and discontinuation^34^ | For Sac/Val, the target dose was achieved in 30% of patients, with discontinuation occurring in 27% of patients |
| **Source:** López-Azor JC et al^35^  **Country:** Spain | Prospective registry in 17 Spanish hospitals including patients with HFrEF who initiated Sac/Val | 527 | 70 [62-78] | 26.9 | 7.1 | Compare the safety of Sac/Val in-hospital initiation with the outpatient initiation^35^ | No significant difference in the incidence of hypotension, worsening renal function, and hyperkalemia between in-hospital and outpatient initiators |
| **Source:** TAROT-HF study^36^  **Country:** Taiwan | Multicenter, retrospective cohort study of patients initiating Sac/Val in 10 hospitals | 1772 | 62.5 (14.7) | 25 | 18 | Report prescription patterns^36^ | 33% of patients received Sac/Val during an HF hospitalization. Only 0.4% of patients initiated Sac/Val at target dose |
| **Source:** EVOLUTION-HF^37^  **Country:** Japan, Sweden, and the US | Multinational longitudinal cohort study of electronic health records in Japan (Medical Data Vision claims registry), Sweden (nationwide administrative registries), and the United States (Optum deidentified Market Clarity Data) of patients initiating medical therapy within 12 months of an HF hospitalization | 29 025 | 71 (12) | 34.7 | 12 | Report prescription patterns^37^ | Mean times from hospitalization to treatment initiation were longer for novel dapagliflozin and Sac/Val than for other therapies. Sac/Val target dose was achieved in 28.2% of patients, while 26.4% discontinued therapy |
| **Source:** Optum EHR database^38^  **Country:** US | The Optum EHR database contains de-identified and aggregated clinical medical administrative data from 85 US healthcare delivery organizations | 3367 | 65.9 (13.1) | 28.7 | 12 | Compare Sac/Val vs ACEi/ARB in naïve patients on the composite of HF hospitalization or emergency room visits^38^ | Incidence rate ratio: 0.87; 95% CI: 0.81-0.94 |
| **Source:** Sentinel Distributed Database^39^  **Country:** US | Patients with HF initiating with Sac/Val or ACEi/ARB | 41 998 | 71.6 (11.8) | 35.4 | 12 | Compare the risk of angioedema among Sac/Val new users vs ACEi/ARB previous users^39^ | Compared with Sac/Val new users, ACEi previous users (HR: 1.98; 95% CI: 1.11-3.53) and ARB previous users (HR: 2.45; 95% CI: 1.36-4.43) experienced an increased risk of angioedema |
| **Source:** OptumLabs Data Warehouse^40,41^  **Country:** US | Retrospective analysis of medical and pharmacy claims | 2244 | 67.6 (12.0) | 31.8 | 18 | Assess the adoption and prescription drug costs of Sac/Val in the first 18 months after Food and Drug Administration approval^40^ | Patients prescribed Sac/Val were younger, more often male, with less comorbidity than those taking ACEi/ARB. Out-of-pocket costs were still high ($40) compared with other HF medication ($2-$3). Overall, 59.1% of patients were adherent to Sac/Val, with half of the nonadherent patients discontinuing treatment within 6 months. |
|  |  | 8291 | 68.2 (12.0) | 32.8 | 6.3 (5.4) | Compare the effectiveness of sacubitril-valsartan and ACEi/ARB on the outcome of all-cause mortality/all-cause hospitalization^41^ | HR: 0.86; 95% CI: 0.81-0.91 |
| **Source:** Kim YS et al^42^  **Country:** Global | Data on adverse events from WHO’s VigiBase, FAERS, and EMA EudraVigilance | 103 038 adverse events from Sac/Val treatment | NA | NA | NA | Compare Sac/Val with other HF medications on incidence of hypotension, renal dysfunction, hyperkalemia, and angioedema^42^ | Hypotension was more frequent for Sac/Val: OR: 11.42 (95% CI: 11.08-11.78). Renal dysfunction, hyperkalemia, and angioedema more frequent with other HF medications |
| **Source:** DISCOVER-ARNi^43^  **Country:** Italy | Multicenter registry of patients with HFrEF initiating Sac/Val | 113 | 65 (11) | 12 | 6 | Eligibility for ICD at 6 months in patients who were ICD-eligible at baseline^43^ | 60% of patients were no more eligible for ICD (EF ≥35% or NYHA class I) |
| **Source:** Monzo L et al ^44^  **Country:** Italy | Patients with HFrEF initiating Sac/Val and implanted with an ICD before Sac/Val availability on the market | 55 | 66.6 (8.9) | 11 | 11 [6-14] | Eligibility for ICD at the end of follow-up in patients ICD-eligible at baseline^44^ | 40% of patients resulted no more eligible for ICD at follow-up |
| **Source:** Nogueira MA et al^45^  **Country:** Portugal | Patients with HFrEF eligible for ICD initiating Sac/Val | 48 | 72.5 (9.8) | 29.2 | 11.3 [9.4-19.6] | Eligibility for ICD at the end of follow-up in patients who were ICD-eligible at baseline^45^ | 56% of patients resulted no more eligible for ICD at follow-up |

Values are mean (standard deviation) or median [interquartile range] if not otherwise specified. ACEi, angiotensin-converting enzyme inhibitor; ARB, angiotensin receptor blocker; CI, confidence interval; CKD, chronic kidney disease; CMR, cardiac magnetic resonance; CPET, cardiopulmonary exercise testing; CV, cardiovascular; EF, ejection fraction; HF, heart failure; HFrEF, heart failure with reduced ejection fraction; HHF, hospitalization for heart failure; HR, hazard ratio; ICD, implantable cardioverter defibrillator; KCCQ-OS, Kansas City Cardiomyopathy Questionnaire Overall Summary Score; LVEDD, left ventricle end-diastolic diameter; LVEDVi, left ventricle end-diastolic volume indexed; MR, mitral regurgitation; MRA, mineralocorticoid receptor antagonist; NA, not available; NT-proBNP, N-terminal pro B-type natriuretic peptide; NYHA, New York Heart Association; OR, odds ratio; PDC, Proportion of Days Covered; QoL, quality of life; RCT, randomized controlled trial; Sac/Val, sacubitril/valsartan; 6MWT, six-minute walking test; UK, United Kingdom; US, United States; WHO, World Health Organization.

**Supplementary Table 2.** Background Medications Used in the Included Studies at the Time of Sacubitril/Valsartan Initiation

| **Source** | **Country** | **Number of patients treated with Sac/Val** | **Enrollment timeframe** | **ACEi/ARB** | **Beta-blocker** | **MRA** | **Loop diuretic** |
| --- | --- | --- | --- | --- | --- | --- | --- |
| PROVE-HF^1-5^ | US | 794 | 2016-2018 | 75.8 | 95.3 | 35.4 | Not reported |
| Cheng Hsin General Hospital^7^ | Taiwan | 437 | 2016-2017 | 73.5 | 83.8 | 70.9 | Not reported |
| ARNi-TR^9^ | Turkey | 704 | 2017-2020 | 72.4 | 83.3 | 73.7 | 90.7 |
| REAL.IT^10^ | Italy | 924 | 2016-2019 | 94.3 | 96.4 | 76.9 | 87.3 |
| CHAMP-HF^12-16^ | US | 746 | 2015-2020 | 65.0 | 96.0 | 47.0 | 72.0 |
| Beltrán P, et al^17^ | Spain | 58 | 2016-2017 | 100.0 | 91.4 | 74.1 | Not reported |
| Mapelli M, et al^18^ | Italy | 96 | 2018-2019 | 98.0 | 99.0 | 72.0 | 79.0 |
| IRRB/23/15^19-21^ | Italy | 134 | 2017-2018 | 81.0 | 93.0 | 82.0 | 83.0 |
| Veterans Affairs Database^23,24^ | US | 1612 | 2015-2017 | 100.0 | 78.7 | 43.4 | 61.7 |
|  |  | 3458 | 2015-2019 | 0.0 | 51.6 | 19.2 | Not reported |
| Swedish HF Registry^27-29^ | Sweden | 1065 | 2017-2021 | 80.0 | 95.2 | 69.0 | 71.0 |
| IMS longitudinal prescription database^30^ | Germany | 12082 | 2016-2017 | 93.1 | 89.7 | 64.1 | 86.4 |
| Badreldin HA et al^31^ | Saudi Arabia | 400 | 2018-2021 | 66.3 | 91.0 | 63.0 | 82.0 |
| GWTG-HF Registry^32,33^ | US | 221 | 2015-2017 | 49.1 | 92.7 | 40.4 | Not reported |
| Savarese G, et al^34^ | US, UK and Sweden | 29546 | 2016-2019 | 78.0 | 90.0 | 52.0 | 30.0 |
| López-Azor JC, et al^35^ | Spain | 527 | 2016-2018 | 95.0 | 91.0 | 63.5 | 77.1 |
| TAROT-HF study^36^ | Taiwan | 1772 | 2017-2018 | 74.2 | 78.0 | 61.3 | 56.1 |
| EVOLUTION-HF^37^ | Japan, Sweden, and the US | 29025 | 2020-2022 | 72.3 | 87.7 | 45.1 | 85.5 |
| Optum EHR database^38^ | US | 3367 | 2015-2019 | 0 | 29.6 | 27.9 | 61.5 |
| OptumLabs Data Warehouse^40^ | US | 2244 | 2016-2016 | 56.1 | 86.5 | 40.1 | 63.7 |
| DISCOVER-ARNi^43^ | Italy | 351 | 2017-2019 | 81.0 | 96.0 | 65.0 | 84.0 |
| Monzo L et al^44^ | Italy | 48 | 2015 | 100.0 | 100.0 | 79.0 | 79.0 |
| Nogueira MA et al^45^ | Portugal | 48 | 2017-2019 | 91.7 | 95.8 | 54.2 | 81.3 |

For all treatments, percentages over the total number of patients are reported. ACEi, angiotensin-converting enzyme inhibitor; ARB, angiotensin receptor blocker; MRA, mineralocorticoid receptor antagonist.

**Supplementary Figure 1.** Quality and Risk of Bias Assessment for the Included Studies

**
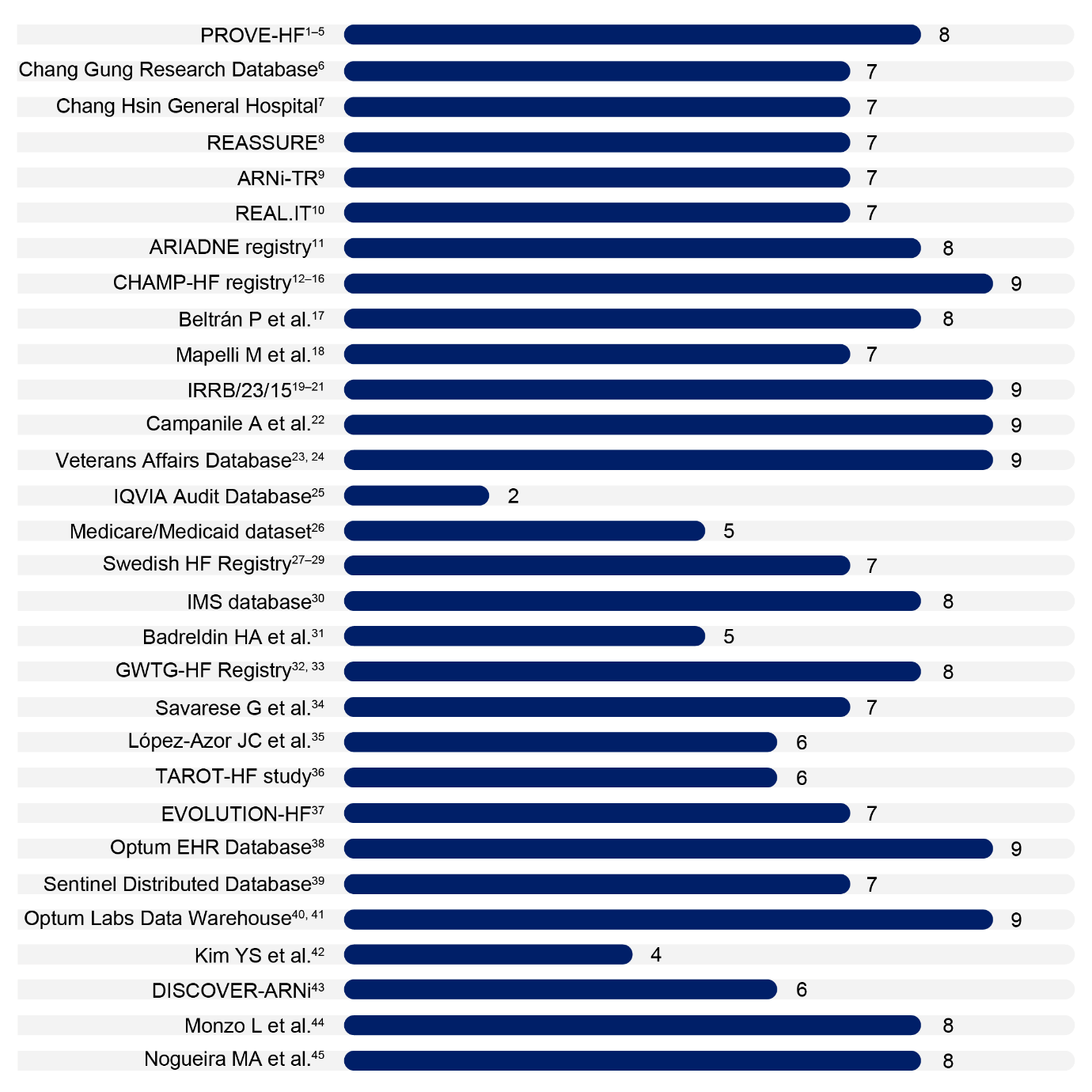
**

**References (All reported in the main manuscript)**

1. Januzzi JL Jr, Prescott MF, Butler J, et al. Association of change in N-terminal pro-B-type natriuretic peptide following initiation of sacubitril-valsartan treatment with cardiac structure and function in patients with heart failure with reduced ejection fraction. *JAMA*. 2019;322(11):1085-1095. doi:10.1001/jama.2019.12821
2. Januzzi JL Jr, Camacho A, Piña IL, et al. Reverse cardiac remodeling and outcome after initiation of sacubitril/valsartan. *Circ Heart Fail*. 2020;13(6):e006946. doi:10.1161/CIRCHEARTFAILURE.119.006946
3. Januzzi JL, Omar AMS, Liu Y, et al. Association between sacubitril/valsartan initiation and mitral regurgitation severity in heart failure with reduced ejection fraction: the PROVE-HF study. *Circulation*. 2022;146(21):1638-1640. doi:10.1161/CIRCULATIONAHA.122.061693
4. Mohebi R, Liu Y, Piña IL, et al. Dose-response to sacubitril/valsartan in patients with heart failure and reduced ejection fraction. *J Am Coll Cardiol*. 2022;80(16):1529-1541. doi:10.1016/j.jacc.2022.08.737
5. Felker GM, Butler J, Ibrahim NE, et al. Implantable cardioverter-defibrillator eligibility after initiation of sacubitril/valsartan in chronic heart failure: insights from PROVE-HF. *Circulation*. 2021;144(2):180-182. doi:10.1161/CIRCULATIONAHA.121.054034
6. Chang PC, Wang CL, Hsiao FC, et al. Sacubitril/valsartan vs. angiotensin receptor inhibition in heart failure: a real-world study in Taiwan. *ESC Heart Fail*. 2020;7(5):3003-3012. doi:10.1002/ehf2.12924
7. Chang HY, Chen KC, Fong MC, et al. Recovery of left ventricular dysfunction after sacubitril/valsartan: predictors and management. *J Cardiol*. 2020;75(3):233-241. doi:10.1016/j.jjcc.2019.08.005
8. Park JJ, Lee SE, Cho HJ, et al. Real-world usage of sacubitril/valsartan in korea: a multi-center, retrospective study. *Int J Heart Fail*. 2022;4(4):193-204. doi:10.36628/ijhf.2022.0015
9. Ekici B, Yaman M, Küçük M, et al. Angiotensin receptor neprilysin inhibitor for patients with heart failure and reduced ejection fraction: Real-world experience from Turkey (ARNi-TR). *Turk Kardiyol Dern Ars*. 2021;49(5):357-367. doi:10.5543/tkda.2021.63099
10. Di Lenarda A, Di Gesaro G, Sarullo FM, et al. Sacubitril/ valsartan in heart failure with reduced ejection fraction: real-world experience from Italy (the REAL.IT study). *J Clin Med*. 2023;12(2):699. Published 2023 Jan 16. doi:10.3390/jcm12020699
11. Maggioni AP, Clark AL, Barrios V, et al. Outcomes with sacubitril/valsartan in outpatients with heart failure and reduced ejection fraction: the ARIADNE registry. *ESC Heart Fail*. 2022;9(6):4209-4218. doi:10.1002/ehf2.14014
12. DeVore AD, Hill CL, Thomas L, et al. Patient, provider, and practice characteristics associated with sacubitril/valsartan use in the United States. *Circ Heart Fail*. 2018;11(9):e005400. doi:10.1161/CIRCHEARTFAILURE.118.005400
13. Khariton Y, Fonarow GC, Arnold SV, et al. Association between sacubitril/valsartan initiation and health status outcomes in heart failure with reduced ejection fraction. *JACC Heart Fail*. 2019;7(11):933-941. doi:10.1016/j.jchf.2019.05.016
14. Thomas M, Khariton Y, Fonarow GC, et al. Association between sacubitril/valsartan initiation and real-world health status trajectories over 18 months in heart failure with reduced ejection fraction. *ESC Heart Fail*. 2021;8(4):2670-2678. doi:10.1002/ehf2.13298
15. Chapman B, Hellkamp AS, Thomas LE, et al. Angiotensin receptor neprilysin inhibition and associated outcomes by race and ethnicity in patients with heart failure with reduced ejection fraction: Data from CHAMP-HF. *J Am Heart Assoc*. Published online June 20, 2022. doi:10.1161/JAHA.121.022889
16. Peri-Okonny PA, Mi X, Khariton Y, et al. Target doses of heart failure medical therapy and blood pressure: insights from the CHAMP-HF registry. *JACC Heart Fail*. 2019;7(4):350-358. doi:10.1016/j.jchf.2018.11.011
17. Beltrán P, Palau P, Domínguez E, et al. Sacubitril/valsartan and short-term changes in the 6-minute walk test: a pilot study. *Int J Cardiol*. 2018;252:136-139. doi:10.1016/j.ijcard.2017.10.074
18. Mapelli M, Mattavelli I, Paolillo S, et al. Effects of sacubitril/valsartan on exercise capacity: a prognostic improvement that starts during uptitration. *Eur J Clin Pharmacol*. 2023;79(9):1173-1184. doi:10.1007/s00228-023-03527-y
19. Giallauria F, Vitale G, Pacileo M, et al. Sacubitril/valsartan improves autonomic function and cardiopulmonary parameters in patients with heart failure with reduced ejection fraction. *J Clin Med*. 2020;9(6):1897. doi:10.3390/jcm9061897
20. Vitale G, Romano G, Di Franco A, et al. Early effects of sacubitril/valsartan on exercise tolerance in patients with heart failure with reduced ejection fraction. *J Clin Med*. 2019;8(2):262. Published 2019 Feb 20. doi:10.3390/jcm8020262
21. Nugara C, Giallauria F, Vitale G, et al. Effects of sacubitril/valsartan on exercise capacity in patients with heart failure with reduced ejection fraction and the role of percentage of delayed enhancement measured by cardiac magnetic resonance in predicting therapeutic response: a multicentre study. *Card Fail Rev*. 2023;9:e07. doi:10.15420/cfr.2022.13
22. Campanile A, Visco V, De Carlo S, et al. Sacubitril/valsartan vs. standard medical therapy on exercise capacity in HFrEF patients. *Life (Basel)*. 2023;13(5):1174. Published 2023 May 12. doi:10.3390/life13051174
23. Mohanty AF, Levitan EB, Dodson JA, et al. Characteristics and healthcare utilization among veterans treated for heart failure with reduced ejection fraction who switched to sacubitril/valsartan. *Circ Heart Fail*. 2019;12(11):e005691. doi:10.1161/CIRCHEARTFAILURE.118.005691
24. Mohanty AF, Levitan EB, King JB, et al. Sacubitril/ valsartan initiation among veterans who are renin-angiotensin-aldosterone system inhibitor naïve with heart failure and reduced ejection fraction. *J Am Heart Assoc*. 2021;10(20):e020474. doi:10.1161/JAHA.120.020474
25. Ozaki AF, Krumholz HM, Mody FV, Jackevicius CA. National trends in the use of sacubitril/valsartan. *J Card Fail*. 2021;27(8):839-847. doi:10.1016/j.cardfail.2021.05.015
26. Sumarsono A, Vaduganathan M, Ajufo E, et al. Contemporary patterns of medicare and medicaid utilization and associated spending on sacubitril/valsartan and ivabradine in heart failure. *JAMA Cardiol*. 2020;5(3):336-339. doi:10.1001/jamacardio.2019.4982
27. Stolfo D, Benson L, Lindberg F, et al. Status and timing of angiotensin receptor-neprilysin inhibitor implementation in patients with heart failure and reduced ejection fraction: Data from the Swedish Heart Failure Registry. *Eur J Heart Fail*. 2024;26(10):2243-2257. doi:10.1002/ejhf.3404
28. Savarese G, Hage C, Benson L, et al. Eligibility for sacubitril/valsartan in heart failure across the ejection fraction spectrum: real-world data from the Swedish Heart Failure Registry. *J Intern Med*. 2021;289(3):369-384. doi:10.1111/joim.13165
29. Fu M, Vedin O, Svennblad B, et al. Implementation of sacubitril/valsartan in Sweden: clinical characteristics, titration patterns, and determinants. *ESC Heart Fail*. 2020;7(6):3633-3643. doi:10.1002/ehf2.12883
30. Wachter R, Fonseca AF, Balas B, et al. Real-world treatment patterns of sacubitril/valsartan: a longitudinal cohort study in Germany. *Eur J Heart Fail*. 2019;21(5):588-597. doi:10.1002/ejhf.1465
31. Badreldin HA, Korayem GB, Alenazy BA, et al. Real-world analysis of integration of sacubitril/valsartan into clinical practice in Saudi Arabia. *Medicine (Baltimore)*. 2023;102(51):e36699. doi:10.1097/MD.0000000000036699
32. Carnicelli AP, Lippmann SJ, Greene SJ, et al. Sacubitril/valsartan initiation and postdischarge adherence among patients hospitalized for heart failure. *J Card Fail*. 2021;27(8):826-836. doi:10.1016/j.cardfail.2021.03.012
33. Carnicelli AP, Li Z, Greiner MA, et al. Sacubitril/ valsartan adherence and postdischarge outcomes among patients hospitalized for heart failure with reduced ejection fraction. *JACC Heart Fail*. 2021;9(12):876-886. doi:10.1016/j.jchf.2021.06.018
34. Savarese G, Bodegard J, Norhammar A, et al. Heart failure drug titration, discontinuation, mortality and heart failure hospitalization risk: a multinational observational study (US, UK and Sweden). *Eur J Heart Fail*. 2021;23(9):1499-1511. doi:10.1002/ejhf.2271
35. López-Azor JC, Vicent L, Valero-Masa MJ, et al. Safety of sacubitril/valsartan initiated during hospitalization: data from a non-selected cohort. *ESC Heart Fail*. 2019;6(6):1161-1166. doi:10.1002/ehf2.12527
36. Lin WY, Chung FP, Liao CT, et al. Treatment with angiotensin receptor neprilysin inhibitor for Taiwan heart failure patients: Rationale and baseline characteristics of the TAROT-HF study. *J Chin Med Assoc*. 2021;84(9):833-841. doi:10.1097/JCMA.0000000000000578
37. Savarese G, Kishi T, Vardeny O, et al. Heart failure drug treatment-inertia, titration, and discontinuation: a multinational observational study (EVOLUTION HF). *JACC Heart Fail*. 2023;11(1):1-14. doi:10.1016/j.jchf.2022.08.009
38. Houchen E, Loefroth E, Schlienger R, et al. Hospitalization rates in patients with heart failure and reduced ejection fraction initiating sacubitril/valsartan or angiotensin-converting enzyme inhibitors/angiotensin receptor blockers: a retrospective cohort study. *Cardiol Ther*. 2022;11(1):113-127. doi:10.1007/s40119-021-00252-4
39. Eworuke E, Welch EC, Haug N, et al. Comparative risk of angioedema with sacubitril-valsartan vs renin-angiotensin-aldosterone inhibitors. *J Am Coll Cardiol*. 2023;81(4):321-331. doi:10.1016/j.jacc.2022.10.033
40. Sangaralingham LR, Sangaralingham SJ, Shah ND, Yao X, Dunlay SM. Adoption of sacubitril/valsartan for the management of patients with heart failure. *Circ Heart Fail*. 2018;11(2):e004302. doi:10.1161/CIRCHEARTFAILURE.117.004302
41. Tan NY, Sangaralingham LR, Sangaralingham SJ, Yao X, Shah ND, Dunlay SM. Comparative effectiveness of sacubitril-valsartan versus ACE/ARB therapy in heart failure with reduced ejection fraction. *JACC Heart Fail*. 2020;8(1):43-54. doi:10.1016/j.jchf.2019.08.003
42. Kim YS, Brar S, D'Albo N, et al. Five years of sacubitril/valsartan-a safety analysis of randomized clinical trials and real-world pharmacovigilance. *Cardiovasc Drugs Ther*. 2022;36(5):915-924. doi:10.1007/s10557-021-07210-1
43. Pastore MC, Mandoli GE, Giannoni A, et al. Sacubitril/valsartan reduces indications for arrhythmic primary prevention in heart failure with reduced ejection fraction: insights from DISCOVER-ARNI, a multicenter Italian register. *Eur Heart J Open*. 2021;2(1):oeab046. Published 2021 Dec 21. doi:10.1093/ehjopen/oeab046
44. Monzo L, Gaudio C, Cicogna F, et al. Impact of sacubitril/valsartan on implantable defibrillator eligibility in heart failure: a real-world experience. *Eur Rev Med Pharmacol Sci*. 2021;25(18):5690-5700. doi:10.26355/eurrev_202109_26788
45. Nogueira MA, Brochado M, Nabais I, Batista É, Matias C, Proença G. Is sacubitril/valsartan able to change the timing for implantation of cardiac devices in heart failure with reduced ejection fraction? *Hearts*. 2022;3(3):88-95. doi:10.3390/hearts3030012
